# Fundamental Concepts of Bipolar and High-Density Surface EMG Understanding and Teaching for Clinical, Occupational, and Sport Applications: Origin, Detection, and Main Errors

**DOI:** 10.3390/s22114150

**Published:** 2022-05-30

**Authors:** Isabella Campanini, Andrea Merlo, Catherine Disselhorst-Klug, Luca Mesin, Silvia Muceli, Roberto Merletti

**Affiliations:** 1 LAM-Motion Analysis Laboratory, Neuromotor and Rehabilitation Department, S. Sebastiano Hospital, Azienda USL-IRCCS di Reggio Emilia, Via Circondaria 29, 42015 Correggio, Italy; isabella.campanini@ausl.re.it (I.C.); or ingmerlo@me.com (A.M.); 2 Merlo Bioengineering, 43121 Parma, Italy; 3 Department of Rehabilitation & Prevention Engineering, Institute of Applied Medical Engineering, RWTH Aachen University, Pauwelsstr. 20, 52074 Aachen, Germany; disselhorst-klug@ame.rwth-aachen.de; 4 Mathematical Biology and Physiology Group, Department of Electronics and Telecommunications, Politecnico di Torino, Corso Duca degli Abruzzi 24, 10129 Turin, Italy; luca.mesin@polito.it; 5 Division of Signal Processing and Biomedical Engineering, Department of Electrical Engineering, Chalmers University of Technology, Hörsalsvägen 11, 41296 Gothenburg, Sweden; muceli@chalmers.se; 6 Laboratory for Engineering of the Neuromuscular System (LISiN), Department of Electronics and Telecommunications, Politecnico di Torino, Corso Duca degli Abruzzi 24, 10129 Turin, Italy

**Keywords:** surface electromyography, surface EMG signal, high-density surface EMG, teaching, electrodes, crosstalk, volume conductor, conduction velocity, modeling

## Abstract

Surface electromyography (sEMG) has been the subject of thousands of scientific articles, but many barriers limit its clinical applications. Previous work has indicated that the lack of time, competence, training, and teaching is the main barrier to the clinical application of sEMG. This work follows up and presents a number of analogies, metaphors, and simulations using physical and mathematical models that provide tools for teaching sEMG detection by means of electrode pairs (1D signals) and electrode grids (2D and 3D signals). The basic mechanisms of sEMG generation are summarized and the features of the sensing system (electrode location, size, interelectrode distance, crosstalk, etc.) are illustrated (mostly by animations) with examples that teachers can use. The most common, as well as some potential, applications are illustrated in the areas of signal presentation, gait analysis, the optimal injection of botulinum toxin, neurorehabilitation, ergonomics, obstetrics, occupational medicine, and sport sciences. The work is primarily focused on correct sEMG detection and on crosstalk. Issues related to the clinical transfer of innovations are also discussed, as well as the need for training new clinical and/or technical operators in the field of sEMG.

## 1. Introduction

Bioelectric signals are the results of the activity of organs that are made of excitable cells, such as the brain, the heart or a muscle. Electrical currents flow through the membranes of these cells and generate action potentials that travel along axons or muscle fibers, triggering other events within or between the cells (e.g., the release of neurotransmitters and the contraction of a muscle fiber). The electric fields generated by these action potentials produce a distribution of electric potential in space, on the skin, that evolves over time, as with a movie.

**Figure 1:**
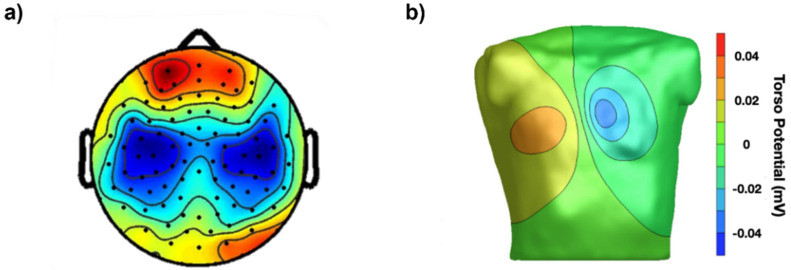
Maps of bioelectric signals: (**a**) example of an instantaneous surface EEG map obtained by a grid of electrodes on the scalp (reproduced from Figure 2 of [1]); (**b**) example of an instantaneous surface ECG map obtained by the simulation of a grid of electrodes on the torso (reproduced from Figure 4 of [2]).

**Figure 2:**
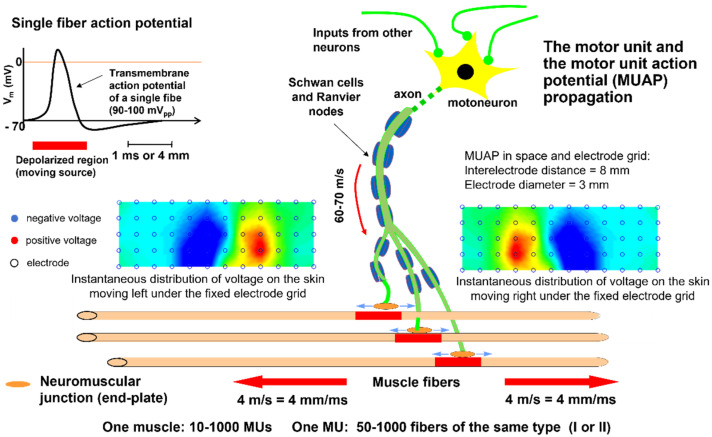
Example of motor unit action potential (MUAP) generation and propagation: electrode grids and maps of the right and left propagating potentials on the skin. Modified from (https://www.robertomerletti.it/en/emg/material/teaching/module5 accessed 29 April 2022, This date applies to all websites and URLs). (See animation in Supplementary Materials, Sup1).

Our eyes cannot see these distributions of voltage or their time evolution on the scalp (electroencephalogram, EEG), on the chest (electrocardiogram, ECG) or on a muscle (surface electromyogram, sEMG), but instruments can and they can visualize them as colored images that we can see and interpret in the same way as thermal images describing temperature distributions over surfaces. To obtain images of voltage distribution, grids of contact points (electrodes) are applied to the chest, scalp or on a muscle, as indicated in Figure 1 and Figure 2. Alternatively, an individual electrode (or a single electrode pair) detects local monopolar voltage with respect to a reference (or a differential voltage), providing a time-varying trace (as with ECG).

Bioelectric signals and images provide an extraordinary opportunity to discover and quantify a wealth of physiological and pathological phenomena that are associated with the organs that generate them. In addition, they provide valuable information to clinicians for functional diagnosis, treatment planning, and the evaluation of the effectiveness of interventions and patient recovery. The role of EEG mapping for studying brain rhythms, the spatial distribution of evoked potentials, epilepsy foci, etc., and the role of ECG mapping in studying atrio-ventricular conduction problems, arrhythmias, etc., are both well known to specialists. The role of sEMG in clinical medicine is less recognized, despite the potential benefits it may offer, mostly because of limited academic teaching [3]. Surface EMG signals are very rich in information about the muscle and neuromuscular system control strategies and can be easily detected by a number of different electrode configurations, with each having advantages, disadvantages, and specific applications.

The purpose of this work is to illustrate in a simple way several fundamental concepts that should be understood and recommendations that should be followed concerning the detection modalities of sEMG (electrode size, interelectrode distance (IED), electrode locations, etc.) in order to (a) be able to extract the information of interest and (b) apply it clinically. The applications of interest range from gait analysis in neurorehabilitation (Section 4.5) to the location of the innervation zone (IZ) for the targeted injection of botulinum toxin (Section 4.3) [4,5], from the estimation of load sharing among muscles [6] to the estimation of muscle fiber conduction velocity to monitor channelopathies and Cushing’s syndrome (Section 2.2) [7,8,9,10], from monitor motor unit (MU) rate modulation and involuntary muscle activity in acute stroke [11,12,13] to study cramps [14], from comparing the ergonomics of chairs (Section 4.6) [15] to detecting when and which muscles are active and which are not [16], along with many other applications in sport and obstetric and rehabilitation medicine.

This work does not aim to present or discuss advances in sEMG technology. Its goal is to illustrate the current technology, in terms of teaching material, tutorials, and consensus papers, concerning the detection and interpretation of sEMG. This work is aimed at teachers, clinical tutors, and students with the objectives of:

(a) overcoming some of the barriers that currently limit the widespread application of sEMG in the clinical world [17,18];
(b) responding to the persistent demand for the simplification of teaching these concepts;
(c) making users aware of the tutorials and teaching materials that are available.

Teaching tools and models are illustrated for this purpose. Some of them were derived from published tutorials [19,20] or modified from the free teaching website https://www.robertomerletti.it/en/emg/material/teaching, where further details can be found. They are presented with the intention of promoting teaching and outlining the improper or incorrect acquisition/interpretation of sEMG.

This paper is structured in six sections with the following purposes: Section 1 introduces the work and its motivations; Section 2 reviews a few basic physiological and technical concepts; Section 3 addresses the issue of sEMG detection using the basic spatial filters; Section 4 addresses the teaching of some clinical applications, focusing on the incorrect use of bipolar and high-density sEMG (HDsEMG); Section 5 and Section 6 discuss the current situation and present conclusions.

## 2. Teaching Basic Physiological and Technical Concepts

Fundamental physiological concepts about sEMG signals, events, and phenomena are reviewed here, together with sEMG modeling techniques that are suitable for teaching, as well as demonstrations.

### 2.1. The Concept of Motor Unit Action Potential (MUAP)

**Figure 3:**
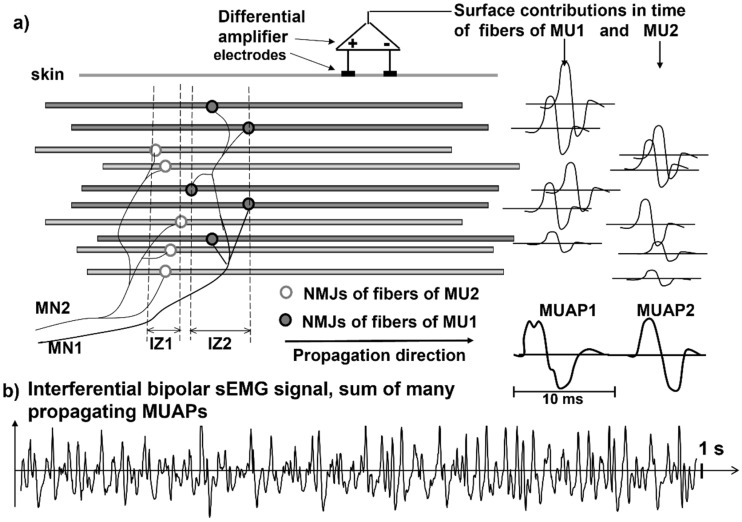
Schematic representation of the fibers of two motor units (MU1 and MU2) that are innervated by two motoneurons (MN1 and MN2) in two innervation zones (IZ1 and IZ2): (**a**) the summation of the skin contributions of the propagating action potentials, as detected by a differential amplifier (bipolar or single differential detection), are depicted as MUAP1 and MUAP2 (observe that the contributions of deeper muscle fibers are smaller than those of the superficial fibers of each MU); (**b**) the interferential signal, which is the summation of the MUAP trains of many active MUs. Modified from Figure 5.1 of [22] with permission.

The sEMG voltage distribution on the skin is the sum of the propagating MU action potentials (MUAPs) produced by the recruited MUs. The MU is the basic muscle building block, consisting of one motor neuron and all of the muscle fibers that it innervates. Every time an axonal action potential reaches the neuromuscular junction (NMJ), acetylcholine is released and a muscle fiber action potential is triggered. Figure 2 and Figure 3 illustrate the concept of MU and the concept of propagating MUAP as a voltage distribution moving on the skin under a fixed grid of electrodes or under a pair of electrodes that are sampling it.

As indicated in Figure 3, each MUAP is the sum of the propagating surface action potentials produced by the single fibers of each MU and the sEMG is the sum of these MUAPs. Force, as well as the features of the sEMG, depends on many factors, including the number of recruited MUs and the discharge rate of each MU, which are both controlled by the central nervous system [21]. The propagation (or conduction) velocity (CV) of the action potentials from the NMJ (or end plate) to the tendon endings depends on the dynamics of the fiber membrane ionic channels.

A fundamental difference between sEMG and ECG or EEG is that electrodes placed on the skin, parallel to the muscle fibers, detect the MUAPs that are propagating bidirectionally from the IZs of the MUs to the muscle–tendon junctions. Since the fibers of an MU are of the same histological type and have about the same diameter, their action potentials travel at the same velocity (CV) along the fibers and their contributions add up, on the skin, as MUAPs that provide a “signature” or “fingerprint” for each MU.

These MUAPs are the basic building blocks of the sEMG. Understanding sEMG implies the understanding of its generation mechanisms. For this reason, our explanations of the physiological concepts focus mostly on single fiber action potentials and/or on single MUAPs whose sum is the sEMG. Figure 3 shows how the contributions of the single fiber action potentials of the MUs add up on the skin to form the MUAPs and how the MUAP contributions add up on the skin to form the sEMG that is detected by one electrode pair.

It should be noted that with progressively stronger voluntary contractions of a muscle, the information about the individual MUAPs becomes progressively more difficult to extract [23].

Although this may seem a simple and straightforward mechanism that is easy to describe and to teach students, a much deeper analysis is needed to fully understand its details. This is necessary to properly pose and answer the following questions: “What information about the MU properties and their central control strategy can be obtained from the sEMG?” and “When can this information be useful?”.

### 2.2. The Concept of Propagating Action Potentials and Their Velocity and Diffusion

#### 2.2.1. Conduction Velocity

**Figure 4:**
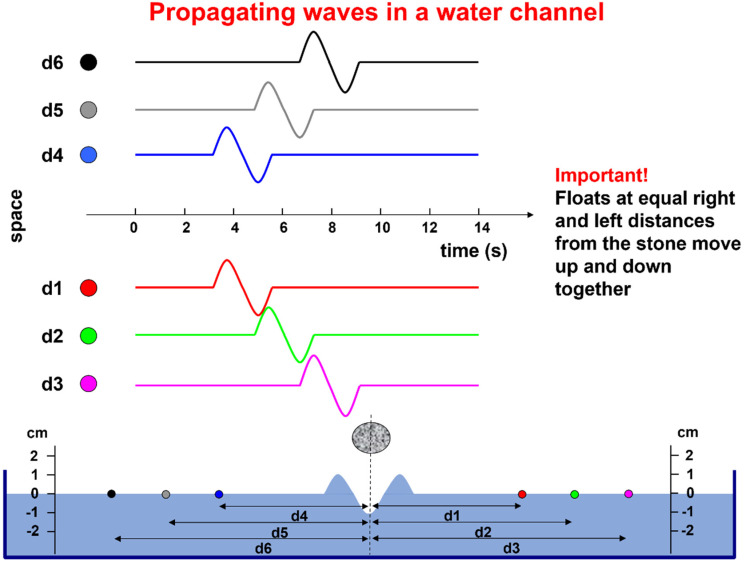
Example of a bidirectional wave generated by dropping a stone in a narrow water channel (representing a single fiber). The floats represent a series of electrodes detecting the wave in subsequent times and in two directions. One pair of floats at equal distances from the stone detect identical waves and their difference is zero. Knowing the distance between floats and the delay between the waves they detect allows for the estimation of the wave propagation velocity. (See animation in Supplementary Materials, Sup2). Courtesy of Dr. Alberto Botter.

For teaching purposes, simple metaphors and examples may be appropriate. One such example is provided in Figure 4. The water channel represents a muscle fiber, the falling stone represents the activation of the NMJ, the wave represents the propagating action potential, the floats represent the electrodes, and their displacement with respect to the resting water level represents the action potential that is detected by each electrode, considering that the wave is as wide as the water channel.

In practice, each muscle fiber can be considered as a line and an action potential moving along it can be approximated by three point-like current sources (a tripole), as described in detail in [24,25,26] and on https://www.robertomerletti.it/en/emg/material/teaching/module9. As is indicated also in Figure 10, the central arrow of the tripole represents positive ions entering the fiber through the open channels and the two lateral arrows represent the positive ions exiting the fiber or negative ions entering it. For the ease of representation and calculation, these ionic currents are modeled as concentrated at three specific points (the tripole).

**Figure 5:**
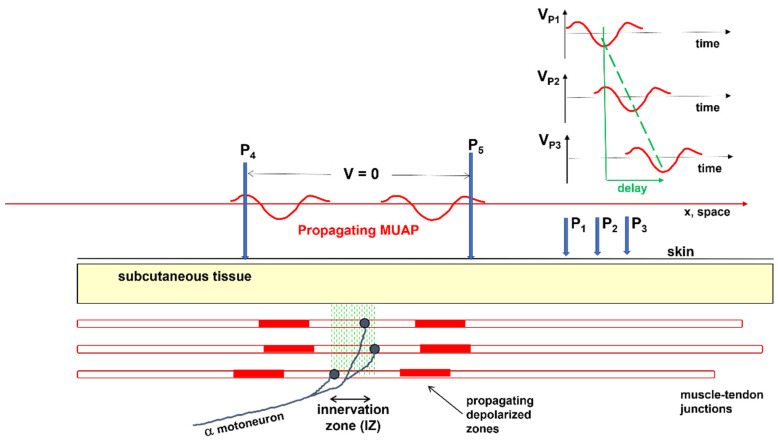
Schematic drawing of the right and left propagation of a motor unit action potential (MUAP) in time and space and the estimation of muscle fiber conduction velocity. When the distance between P1 and P3 is 20 mm and the delay is 5 ms, the muscle fiber conduction velocity is 20/5 = 4 mm/ms = 4 m/s. P1, P2, and P3 must be on the same side of the innervation zone. The signals detected by P1, P2, and P3 are called “monopolar”. The signals detected between P1–P2, P2–P3, and P1–P3 are called “bipolar” or “single differential (SD)”. The SD signal detected between P4 and P5 is near zero because of the symmetrical position of P4 and P5 with respect to the innervation zone. Modified from https://www.robertomerletti.it/en/emg/material/teaching/module5. (See animation in Supplementary Materials, Sup3).

Figure 5 shows the two MUAPs propagating to the right and left of the IZ of a three-fiber motor unit. The volume conductor is layered (muscle, subcutaneous tissues, and skin). The red bars represent the single-fiber depolarized regions. Three detection points (P1, P2, and P3) are placed on the skin to detect the MUAP traveling to the right. The animation (Supplementary Materials, Sup3) shows the concept of propagation in time and space, as well as how the MUAP CV can be estimated by placing a series of electrodes along the muscle fiber direction. The electrode pair P4 and P5 detects a differential signal near zero because of the symmetrical position of P4 and P5 with respect to the IZ.

A single fiber action potential, or MUAP, produces a two-dimensional wave of electric potential over the skin that moves along the muscle (Figure 2 and Figure 5). To summarize, MUAPs produce waves of monopolar potential across the skin over a muscle. Similar to water waves, these potentials travel on the skin from the region above the NMJ toward the tendons. During travel, they are detected by the electrodes. Electrode pairs are connected to a probe that contains a circuit, called a differential amplifier, that computes the difference between the two potentials that are present, instant by instant, below the two electrodes. Since the sEMG potentials are traveling along muscle fibers, the wave of potential of each MU reaches the electrodes at different times. This process is illustrated in Figure 5 and Figures 7–9.

#### 2.2.2. The Concepts of Volume Conductor and Crosstalk

**Figure 6:**
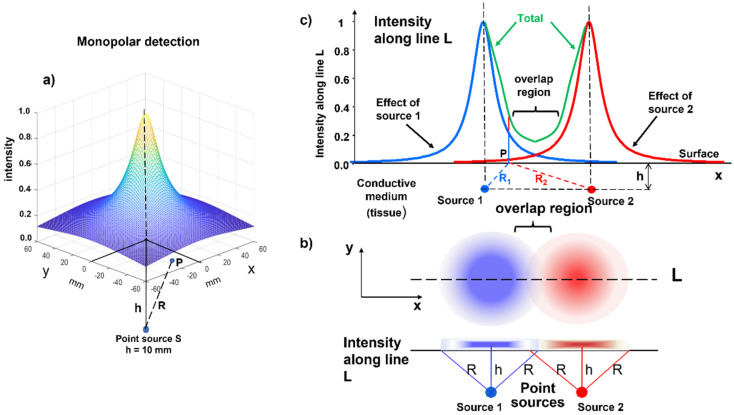
Overlapping of the distributions of surface intensities: (**a**) distribution of intensity generated by a point source of light (or a source of electric potential) at depth h below the surface; (**b**) partially overlapping distributions of light intensities generated by two sources at depth h; (**c**) profiles of partially overlapping light intensities on the surface and along the line L. The same considerations apply to point sources (S_1_ and S_2_) of electric potential whose surface maps extend along x and y, as indicated in (**b**). The intensity I_P_ in point P on the surface is inversely proportional to the distance R_1_ between S_1_ and P and to the distance R_2_ between S_2_ and P, i.e., the voltage in P is V_P_ = kS_1_/R_1_ + kS_2_/R_2_, where k is a proportionality constant that accounts for the medium properties.

We now look at the skin from above as the surface of a liquid containing light sources. For simplicity, we now consider a point-like light source that is at some depth in this liquid, which causes a halo to appear on the surface. In the same way, the source of an electric field at some depth below the skin produces a voltage distribution on the skin surface due to diffusion in the “conductive volume” (or “volume conductor”) of the tissue, as indicated in Figure 6a. This effect is known as the “spatial low-pass filter effect”, which is introduced by the volume conductor and means that MUs that have a greater distance from the detection system generate smaller, but spatially more extended, potential distributions.

Two point-like light sources (one blue and one red) produce the two partially overlapping halos, as represented in Figure 6b,c. The same happens for MUAPs belonging to different muscles: their surface representations can reach nearby muscles. This phenomenon is called “crosstalk”, i.e., the presence of potentials on a target muscle that are produced by a different muscle. This may happen because of the blurring effect that is produced by the subcutaneous tissues and by a further phenomenon called the end-of-fiber (EOF) effect, which is discussed later. The following section explains the detection modalities that have been developed to partially compensate for this blurring effect, thus providing “spatial selectivity” to the sEMG measurement, which is often a pre-requisite for clinical application.

### 2.3. Teaching Differential/Bipolar Detection, Innervation Zones, and the End-of-Fiber Effect

**Figure 7:**
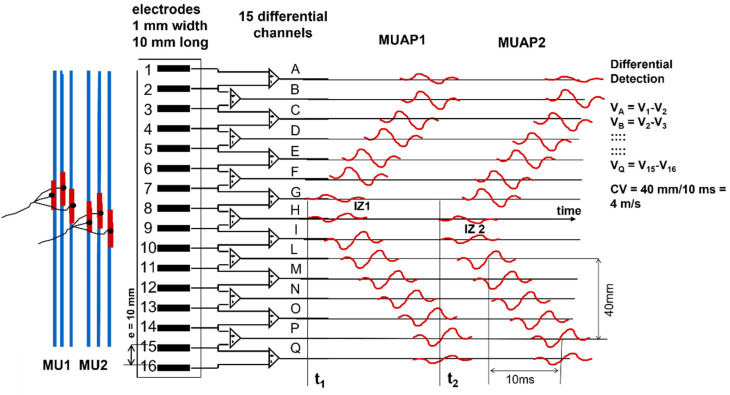
Schematic drawing of the propagation of two motor unit action potentials (MUAPs) under a linear electrode array that is aligned with the fiber direction. MU1 discharges at time t_1_ and MU2 discharges at time t_2_: example of the estimation of the innervation zones and the calculation of the muscle fiber conduction velocity (CV). (See animation in Supplementary Materials, Sup4). Reproduced from (https://www.robertomerletti.it/en/emg/material/teaching/module6).

The electrical image describing the instantaneous distribution of the sEMG voltage is called “monopolar” when the voltage of each point is referred to a remote reference taken as zero (i.e., the resting water level in Figure 4). This image is “sampled” in a number of points in space (electrodes), whose voltages can be combined and manipulated to achieve specific purposes. Figure 7 shows a set of the simplest of these manipulations, called “spatial filters” (see Section 2.3.1 and Section 2.3.2). The time-varying image generated by the firing of MU1 and MU2 is sampled in space, along a line, by a 16-electrode linear array and the differences **V_A_ = V_1_ − V_2_**, **V_B_ = V_2_ − V_3_**, … up to **V_Q_ = V_15_ − V_16_** are presented as output signals. Note that these differences are calculated by the 15 differential amplifiers that compute **V_+_ − V_−_**. If the linear electrode array were inverted (with Electrode 16 at the top and Electrode 1 at the bottom), the polarity of all signals would be inverted. Note that the two MUs have different IZs, located below Electrodes 7–8 (Channel G) and Electrodes 8–9 (Channel H), and their fibers terminate under the two ends of the linear electrode array. Therefore, it is possible to estimate the MU fiber length, as well as the CVs and the IZs. Note, for MU1, the lack of propagation in the action potentials that are detected by Amplifier A (compared to B) and Amplifier Q (compared to P). This lack of propagation indicates the end of the fibers (better defined later in this subsection) and, therefore, their length spans the distance between Amplifiers B and P. Additionally, note that the channels on one side of the IZ have a polarity while those on the other side have the opposite polarity. The amplitudes of the single differential (SD) signals detected above the IZs are near zero.

Note that signals from double (or multiple) IED values may be obtained either by taking the difference between the corresponding monopolar signals or by adding adjacent SD signals, since **V_1_ − V_3_ = (V_1_ − V_2_) + (V_2_ − V_3_)**. Double differential (DD) signals can be obtained from the difference between adjacent SD signals.

These are useful properties of differential detection and allow for the easy identification of the IZ of an MU as the region where the action potentials change polarity. This information can have clinical applications (see Section 4.4).

**Figure 8:**
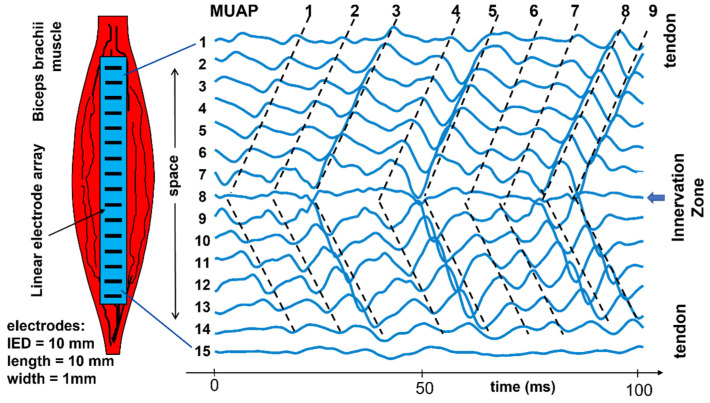
Example of experimental sEMG signals recorded with 15 single differential channels from a biceps brachii. We can see the “signatures” of at least 9 MUs, locate their innervation zones, and estimate their length and conduction velocity (CV). The slopes of the dashed lines are the CVs of the motor unit action potentials (MUAPs). This piece of information cannot be obtained by one pair of electrodes and requires detection by an array of electrodes placed along the muscle fiber direction, as in Figure 7. IED = interelectrode distance. Modified from https://www.robertomerletti.it/en/emg/material/teaching/module6.

**Figure 9:**
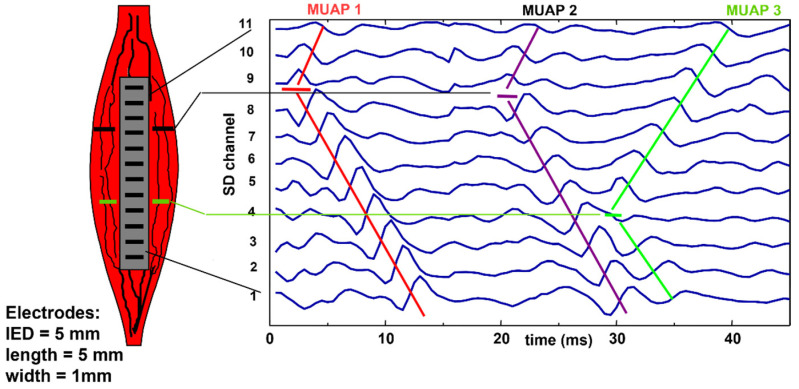
Example of experimental sEMG signals recorded with a 12-electrode array providing 11 single differential (SD) channels from a biceps brachii. In the 45-ms window, we can see the “signatures” of three MUs and locate their innervation zones. In this case, global conduction velocity (CV) can only be estimated in the proximal and distal regions of the muscle. In the middle region (Channels 4 to 8), some motor unit action potentials (MUAPs) propagate upward and some downward. The concept of global, or mean, muscle fiber CV is meaningless in this region and can only be estimated for individual MUs after extracting their MUAPs by means of sEMG decomposition [23]. IED = interelectrode distance. Reproduced from https://www.robertomerletti.it/en/emg/material/teaching/module6.

Figure 8 and Figure 9 depict experimental differential recordings from two biceps brachii, both obtained with linear electrode arrays. Nine MUAPs can be identified in Figure 8 within a 100-ms time window. It is evident that all of the detected MUs are innervated under Channel 8 and have about the same length. Minor differences between their conduction velocities cannot be detected visually and require calculations using software. Figure 9 shows three MUAPs generated by different MUs within a 45-ms time window. Two MUs are innervated between Channels 8 and 9 and one is innervated under Channel 4. Note that (a) the MU fibers are longer than the array and (b) the MUAP propagation is monodirectional upward from Channel 9 to 11 and downward from Channel 3 to 1 and bidirectional between Channels 8 and 4 (downward for MU1 and MU2, upward for MU3). The average value of the muscle fiber CV (global CV) cannot be estimated using Channels 4 to 8. The decomposition of the sEMG into the constituent MUAP trains is necessary to estimate the CVs of individual MUs [23].

**Figure 10:**
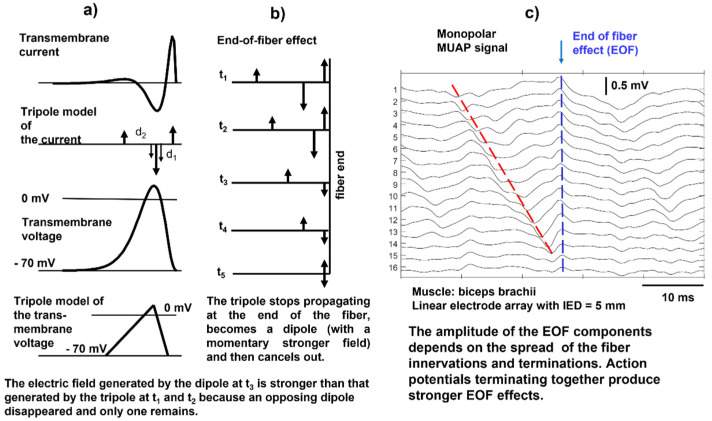
(**a**) Schematic representation of the transmembrane current as two opposite dipoles (a tripole) of current, which implies the representation of the action potential as a triangular waveform; (**b**) extinction of the tripole at the end of the fiber, where the dipole d_1_ shrinks (at times t_1_ and t_2_) and disappears (at time t_3_), leaving dipole d_2_ whose field is no longer partially canceled by d_1_. As d_2_ shrinks and disappears (at times t_4_ and t_5_, respectively), the surface potential disappears; (**c**) example of a real monopolar motor unit action potential (MUAP) whose propagation phase is followed by a non-propagating transient due to the end-of-fiber (EOF) effect. This transient is partially removed by bipolar (SD) detection. In general, the sEMG signal includes propagating and non-propagating components, whose presence requires caution in selecting the signal segment to use for the estimation of muscle fiber CV. IED, interelectrode distance. Modified from https://www.robertomerletti.it/en/emg/material/teaching/module5.

**Figure 11:**
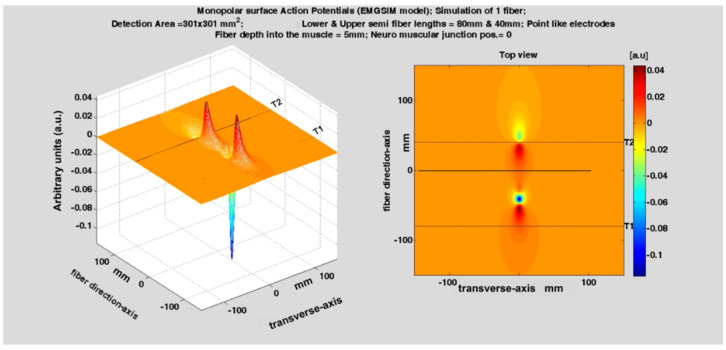
Slow-motion simulation of the generation, propagation, and extinction of an action potential produced by a single fiber whose neuromuscular junction is not in the middle of the fiber. Observe the two end-of-fiber effects taking place at different times at the fiber-tendon junctions T1 and T2. They generate two sharp non-propagating transients in the sEMG signal. For a motor unit, the end-of fiber effect(s) may be smaller and less sharp, depending on the scatter of the fiber terminations. (See animation in Supplementary Materials, Sup5). Reproduced from https://www.robertomerletti.it/en/emg/material/teaching/module9.

When the action potential reaches the end of a muscle fiber, it stops generating a transient signal; this is referred to as the end-of-fiber (EOF) effect, which is described in detail in https://www.robertomerletti.it/en/emg/material/teaching/module5 and https://www.robertomerletti.it/en/emg/material/teaching/module9. This phenomenon is modeled by describing the transmembrane current distribution with a tripole that is made of two opposing dipoles (d_1_ and d_2_), as indicated in Figure 10a. When the tripole reaches the end of the fiber, the first dipole shrinks (Figure 10b, times t_1_ and t_2_) and disappears (Figure 10b, time t_3_), leaving the second dipole d_2_ whose field is stronger because it is no longer partially canceled by d_1_. In turn, d_2_ shrinks (Figure 10b, time t_4_) and disappears (Figure 10b, time t_5_). This model explains the presence of non-traveling waves in the monopolar signals, as indicated in Figure 10c, but the exact mechanism is not known. Non-propagating signal components are often observed in monopolar sEMG signals and are reduced by bipolar (SD) detection, as well as other “common mode” signals. An example of the EOF effect is provided in the simulation video of Figure 11.

#### 2.3.1. Teaching Action Potentials through Modeling

Despite their many limitations, models of sEMG are powerful teaching tools because they can show, with some approximation, the effects that changing individual fiber (or MU) parameters have on the sEMG signal [24,25,27,28]. Additional examples of this use of models for teaching muscle electrophysiology and interpreting sEMG features are provided in https://www.robertomerletti.it/en/emg/material/teaching/module9.

**Figure 12:**
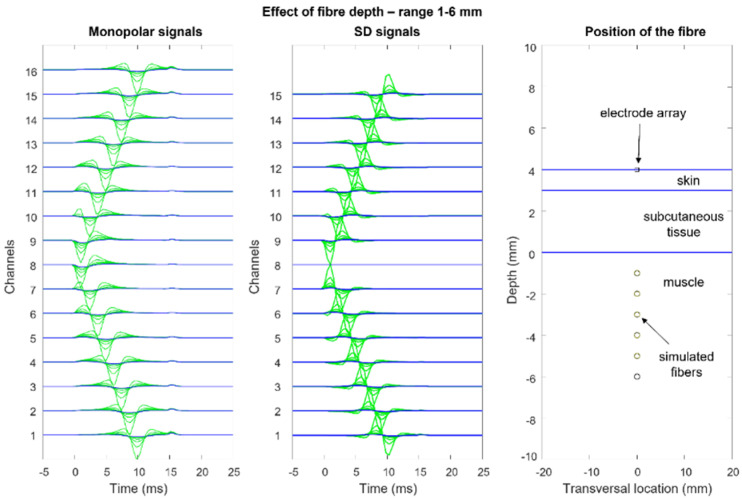
Effects of fiber depth within the muscle: skin thickness = 1 mm, subcutaneous (fat) tissue thickness = 3 mm, interelectrode distance = 5 mm. Observe how, in the monopolar recording, the end-of-fiber effect decreases slowly and becomes greater than the propagating component for depths near 8 mm within the muscle. This contributes to the crosstalk signal that is present on nearby muscles. (See animation in Supplementary Materials, Sup6). Reproduced from https://www.robertomerletti.it/en/emg/material/teaching/module9.

**Figure 13:**
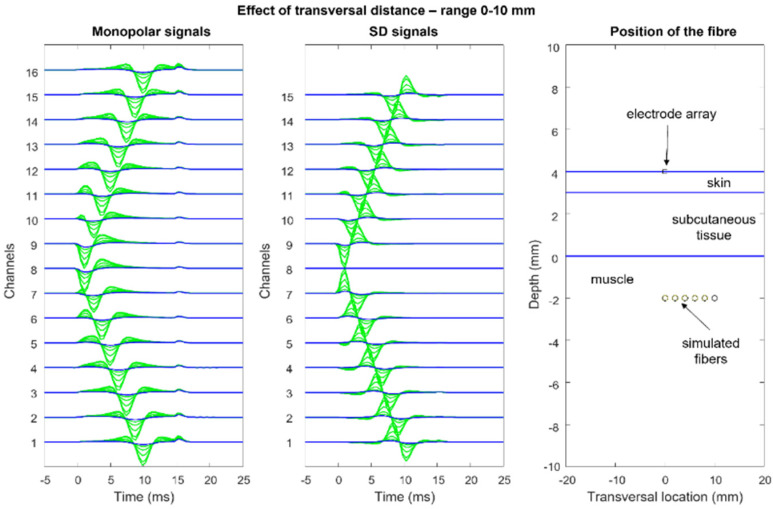
Effects of lateral displacement (transversal distance) between the electrode array and the fiber: same conditions as in Figure 12. Observe that the end-of-fiber effect is visible for distances of greater than 10–15 mm, where it becomes larger than the propagating component. This contributes to crosstalk between muscles that is still detectable at lateral distances of 20–30 mm from the edge of a muscle, mostly as a non-propagating component. (See animation in Supplementary Materials, Sup7). Reproduced from https://www.robertomerletti.it/en/emg/material/teaching/module9.

**Figure 14:**
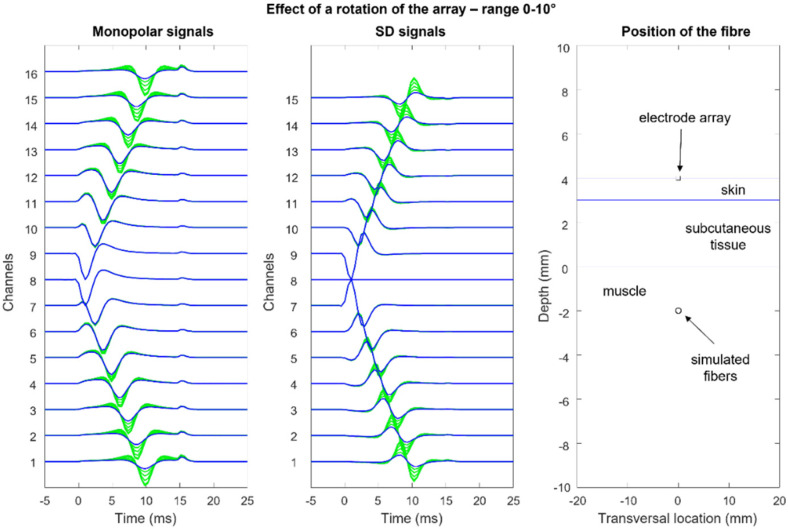
Effects of the rotation of the fiber with respect to the array (or vice versa): the rotation center is the center of the fiber that coincides with that of the array and with the innervation zone (Channel 8). Observe how the detected propagating action potential decreases as it moves along the fiber. This is because the source moves away from the electrodes. The case of a fiber inclined and displaced laterally with respect to the electrode array (not presented) is frequent for muscles that have a fan-like or pinnate structure. More information is provided in [29]. (See animation in Supplementary Materials, Sup8). Reproduced from https://www.robertomerletti.it/en/emg/material/teaching/module9.

**Figure 15:**
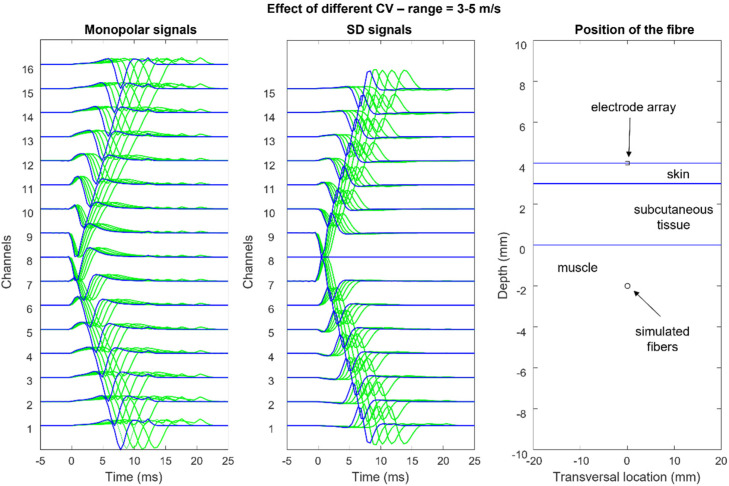
Effects of changing muscle fiber conduction velocity (CV). Observe that the width of the action potential and the end-of-fiber effect become narrower and the slope of the pattern increases as the CV increases. The observed changes affect both the single sEMG channels and the array of signals. When a single channel is recorded, the effects are not detectable. (See animation in Supplementary Materials, Sup9). Reproduced from https://www.robertomerletti.it/en/emg/material/teaching/module9.

Figure 12, Figure 13, Figure 14 and Figure 15 show the simulation of an action potential propagating along a single fiber that is innervated in the middle and detected by a 16-point-like-electrode array with an IED of 5 mm. The monopolar and SD signals are presented. The effects of fiber depth, lateral displacement with respect to the electrode array, the misalignment angle between the fiber and the electrode array, and conduction velocity are depicted. The effects are about the same for an MUAP when the IZ of the MU is narrow.

According to the model, the decrement of sEMG amplitude (root mean square, RMS) with increasing depth within the muscle is similar to that due to the increasing thickness of subcutaneous tissue. Experimental findings from obese subjects have indicated that sEMG is still detectable with subcutaneous tissue thicknesses of 20–30 mm [30]. The lowering of the electrode–skin interface noise may increase detectability at greater distances in the future [31].

Simulations of pathological fibers and MU alterations are lacking in the literature and provide an interesting research field for clinical application, the improvement of the available models, and application in the teaching and clinical interpretation of sEMG signals. A review of the use of sEMG in neuromuscular pathologies is provided by Hogrel [32].

#### 2.3.2. Spatial Filters

**Figure 16:**
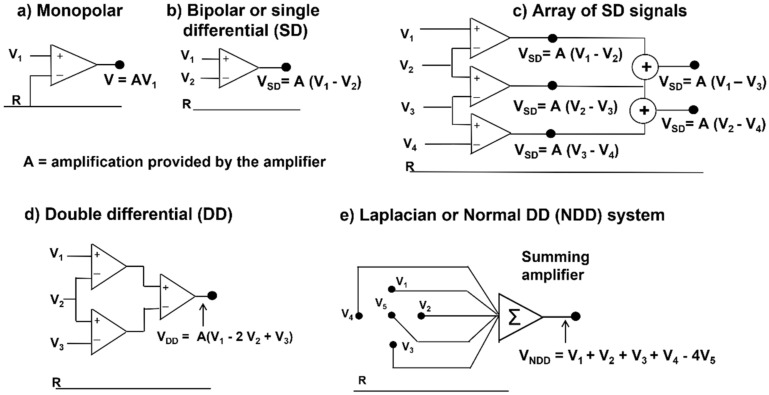
Electrode configurations or “montages”: (**a**) monopolar (any combination or spatial filter can be obtained by a grid of monopolar signals); (**b**) bipolar or single differential (SD); (**c**) array of SD signals; (**d**) double differential (DD); (**e**) normal (perpendicular) DD (NDD) or Laplacian configuration. Other configurations are possible. The reference electrode R must be on a region with no sEMG activity and away from the muscle of interest. It should not be on the same muscle. The NDD system is widely used in EEG because of its selectivity. Some recently developed systems do not need a reference electrode. A is the amplification or gain of the differential amplifier. Modified from https://www.robertomerletti.it/en/emg/material/teaching/module6.

The only potential map that is generated on the skin by a biological signal is the monopolar map. Other maps may be obtained using analogue or numerical combinations of monopolar electrode potentials, as indicated in Figure 16. These combinations, or montages, are usually “differential” and have different properties, with the main difference being the reduction in the detection volume, i.e., greater spatial selectivity. However, “low-pass spatial filters” may be obtained using larger electrodes or averaging blocks of monopolar signals to simulate larger electrodes.

### 2.4. Two-Dimensional (2D) sEMG Representation and sEMG Images

With respect to linear electrode arrays, bi-dimensional electrode arrays or grids provide additional information in the muscle fiber transversal direction and allows for the better discrimination between MU “signatures” and orientation, thereby facilitating the decomposition of the sEMG into the constituent MUAPs, as well as the localization of the active MUs in space and the size of the MU territory (i.e., the spatial distribution of the muscle fibers belonging to an MU).

**Figure 17:**
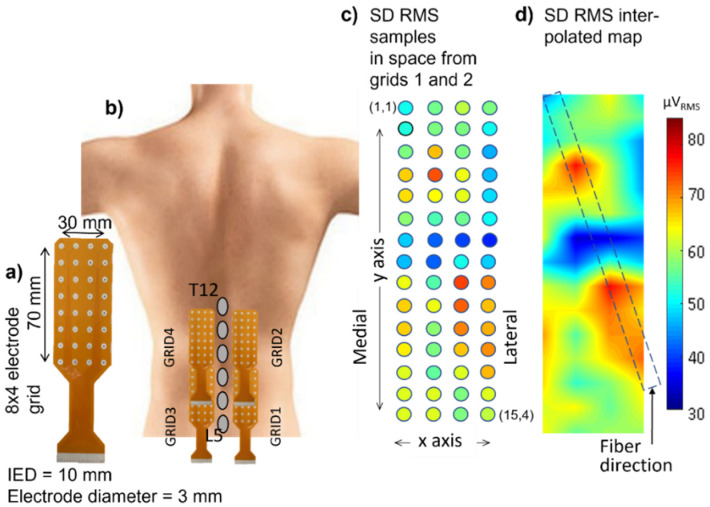
Example of sEMG distribution on the erector spinae of a healthy subject: (**a**) grid of 8 × 4 electrodes (electrode diameter = 3 mm; center-to-center interelectrode distance IED = 10 mm). (**b**) two such grids are applied on each side of the spine to produce an array of 15 × 4 single differential signals whose root mean square (RMS) value (over a 1s epoch) is depicted in (**c**), according to the color scale; (**d**) interpolated image, obtained from (**c**), of the RMS distribution with an indication of the orientation of the fibers. The blue area in the middle corresponds to the innervation zone of the superficial motor units.

Figure 17a shows a 2D grid of 8 × 4 electrodes. Two of these grids are applied to the right erector spinae and two are applied to the left, as in Figure 17b,c, which shows the values (see the color scale) of the RMS of the differential voltage that is detected by each longitudinal pair of electrodes of the two grids placed from proximal to distal, on the right side, over a time window of 1 s. Figure 17d shows the interpolated version of the map and the direction of the fibers. The orientation of the fibers is outlined. It is also evident that the quality (and correctness) of the interpolated map is strongly dependent on the IED. In the case of sEMG images, an IED of 10 mm is a borderline value [33] and provides an image reconstruction that has minor inaccuracies (see Section 4).

The concepts presented in this section raise a number of practical questions, such as “What should the size (i.e., the electrical contact area) of the electrodes and the IED be to allow for the correct reconstruction of a sampled image by interpolation?”, “Are there optimal values for the size and IED for maximal information extraction?”, “How big should an electrode grid be?”, “Where should it be located?”, “What are the advantages and disadvantages of monopolar versus SD images?”, and “When is a single electrode pair preferable to HDsEMG?”. These and many other issues are addressed in Section 3 and Section 4 and in sEMG textbooks and articles [22,33,34] (see https://www.robertomerletti.it/en/emg/material/books/ for a more complete list), which provide a comprehensive mathematical analysis of these problems. Section 3 and Section 4 attempt to answer these questions in a simple way.

## 3. The Role of Electrode Size and Interelectrode Distance in Bipolar sEMG

Electrodes that are applied to the skin are not point-like, as is often assumed in modeling. From the theoretical signal processing point of view, the full information that is contained in the superficial distribution of EMG potential can only be obtained when proper sampling in space is performed (and when spatial aliasing and smoothing are avoided, see [33] for more details). This implies the need for small electrode sizes and small IEDs (a) to avoid the substantial filtering that is introduced by the transfer function of the electrode pair and (b) to reduce crosstalk. In turn, this implies the need for the selective detection of the most superficial MUs.

However, in many clinical applications, only a portion of this information is useful and greater detection volumes may be desirable, which provide larger signals at the price of lower selectivity and possible crosstalk from nearby muscles [35,36]. This also applies to single electrode pairs, in which small EEG electrodes (e.g., http://www.gereonics.com/eegelectrodes.html may be preferable to the larger pediatric ECG electrodes that are often used. Commercially available active sEMG sensors that carry two parallel silver bars, 10 mm long and 10 mm apart, also provide a good solution (e.g., https://delsys.com/sensors.

A pair of large electrodes (diameter = 10 mm) and a large IED (e.g., 20 mm) introduce the attenuation of the highest harmonics in the signal (see Figures 2.5 and 3.11 of [35]), as explained in the following subsections. For most clinical applications of bipolar sEMG, this low-pass filtering is acceptable. Conversely, the reduced selectivity, i.e., the risk of crosstalk, may be critical and lead to misinterpretations. Thus, miniaturized electrodes should be used when assessing muscles with small cross-sectional areas, such as forearm muscles and children’s muscles.

In sport applications, sEMG can be used to compute the so-called localized myoelectric manifestations of muscle fatigue. Since this indicator is based on the spectral features of the signals, the filtering effect of the detection system (i.e., electrode size and IED) cannot be ignored. Users must be aware that their results depend on the electrode configuration and cannot be compared to those obtained using different electrode geometries, e.g., in the literature. The user must also be aware that the electrode size is the electrical contact area (possibly mediated by gel) and does not include the surface of the external adhesive disks that keep the electrode in place when present.

### 3.1. Electrode Size and Its Filtering Effect

**Figure 18:**
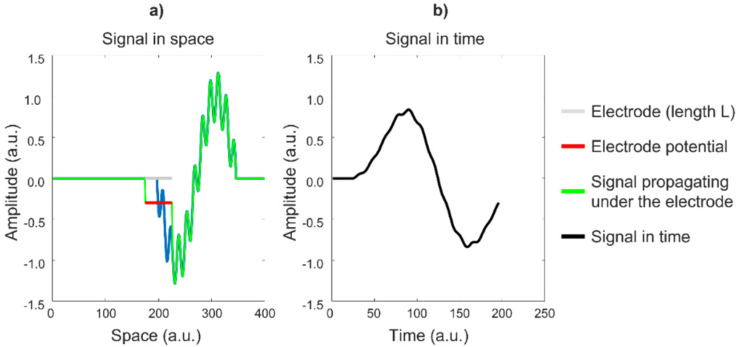
Effects of electrode length on a signal: (**a**) the green signal propagates in space under the gray fixed electrode of length L, generating the instantaneous signal indicated by the red bar. The signal evolves in time, as depicted in panel (**b**). (See animation in Supplementary Materials, Sup10). Courtesy of Dr. Alberto Botter.

**Figure 19:**
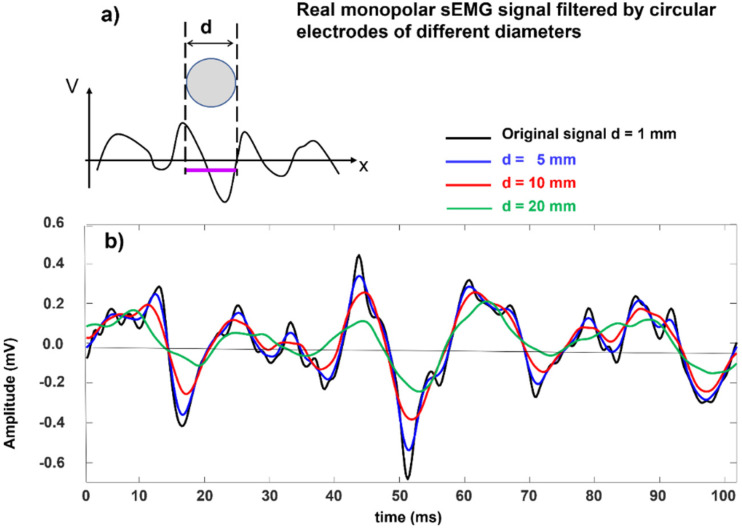
Electrode filtering of a monopolar experimental sEMG signal. (**a**) moving average performed by a round electrode over a propagating monodimensional signal, (**b**) a signal collected using a pin electrode is filtered by the transfer function of electrodes of different sizes (in one dimension only), producing a number of outputs that correspond to different electrode diameters. The signal is low-pass filtered and attenuated because only the filtering effect of the different electrode sizes are accounted for. In practice, a larger electrode covers more fibers, so the amplitude may not be reduced (it may actually increase) but the spectral alteration due to filtering remains and alters the amplitude and spectral features of the signal. Modified from https://www.robertomerletti.it/en/emg/material/teaching/module9.

An electrode has a highly conductive surface, which forces all of the points below it to have the same voltage. This voltage is approximately the mean value of the voltages at the points below the electrode surface (without the electrode). This averaging effect is a form of low-pass filtering, which reduces the “sharpness”, or high-frequency content, of the detected voltage. To understand the relevance of this effect using a simple example, consider the animation in Figure 18a, in which a signal propagates under an electrode of length L, which provides the average value of the signal over L in the direction of propagation versus time (Figure 18b). The electrode attenuates and smooths the signal, thereby introducing low-pass filtering. Observe the cancelation of the periodic signal, which has a period in space (wavelength) that is equal to L/2. Figure 19 shows this effect on a real monopolar sEMG signal. In practice, since a larger electrode increases the detection volume, the amplitude may not be reduced (it may actually increase because a larger number of MUs is covered by the electrode) but the spectral alteration (smoothing) remains.

As indicated in the literature [33], sEMG electrodes with diameters of up to 3 mm are acceptable, while diameters greater than 5 mm modify the shape and the spectral features of the MUAPs of the most superficial Mus, thereby making these MUAPs more similar to those of deeper MUs.

Again, it is clear that electrodes that have diameters of 5 mm are barely acceptable, while electrodes that have diameters of ≥10 mm introduce substantial filtering and modify the waveshape, amplitude, and spectral features of the MUAPs and, with that, the interferential sEMG signal, particularly in lean subjects with many MUs that are located close to the skin surface (see Section 3.2). Nevertheless, these alterations may be acceptable or irrelevant for a number of applications whose objectives are little affected by these factors, such as the analysis of gait or studies on coordination with one, properly positioned, electrode pair per muscle.

### 3.2. Interelectrode Distance and Its Filtering Effect

**Figure 20:**
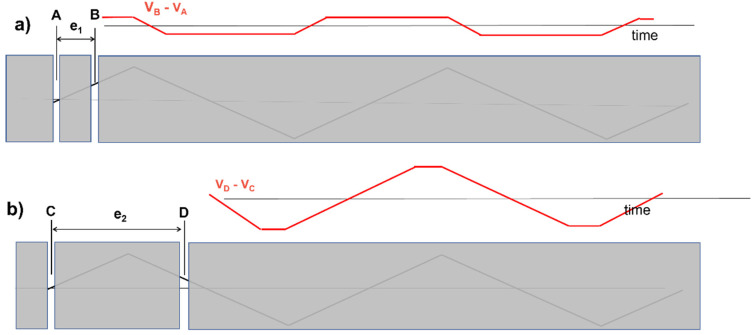
Concept of spatial filtering that is introduced by bipolar (SD) detection. Panels (**a**,**b**) show how the same triangular voltage profile propagating under an electrode pair can produce very different SD signals, depending on the interelectrode distance (IED). The same happens for the sEMG. To avoid this effect in sEMG detection, IEDs should be ≤5 mm (but values of up to 8–10 mm introduce signal alterations that are acceptable for most applications). This is particularly important for detection using electrode grids. It is less important for clinical bipolar detection, as long as the same electrode size, IED, and location are used in subsequent measurements for comparing sEMG features, for example, in pre- and post-treatment or intervention. (See animation in Supplementary Materials, Sup11). Reproduced from https://www.robertomerletti.it/en/emg/material/teaching/module9.

The ubiquitous differential detection (SD) provides the voltage difference (versus time) between two electrodes that are spaced by an IED in the fiber direction. Figure 20 demonstrates how the IED affects the detected SD waveform and, consequently, the sEMG features. A simple triangular waveform propagating under the two electrodes systems A–B, spaced by an IED of e_1_, and C–D, spaced by an IED of e_2_, is provided as a teaching example. The results in time are very different. If e_2_ were equal to the wavelength of the propagating signal, the output in time would be zero at all times, thereby generating an artificial “dip” in the amplitude and power spectrum of the time signal for that particular frequency. For this reason, the IED must be smaller than half of the shortest wavelength that contributes to the sEMG signal, which is about 10 mm (corresponding to 100 cycles/m) for MUs that are close to the skin surface [33]. According to mathematical theory (Nyquist sampling theorem, not discussed here), the spacing between two electrodes should therefore be less than 5 mm. However, due to the spatial low-pass filtering effect of the volume conductor, the minimum wavelength that contributes to the potential distribution is muscle- and subject-dependent. Deeper muscles show skin potential distributions with longer wavelengths. The potential distributions of people with a higher body mass index have longer wavelengths than those of people with a lower body mass index. Therefore, in practice, values of up to 8–10 mm may be acceptable, with minor signal alterations because of the small and long “tails” of the sEMG power spectrum. The effects of electrode size could be added to the simulation in Figure 20 using wider slots and taking the average value of the signal within each slot. See also Figure 18.

In clinical practice, SD (bipolar) sEMG detection is often performed using electrodes that have diameters of 10 mm or more and an IED of 20 mm or more. The combined effect of these values has been discussed in a tutorial and a textbook (see Figures 13 and 14 of [19,35]). Of course, a signal may be obtained using any combination of electrode size and IED and two signals that are detected by different detection systems may look visually alike but have different spectral features because of the electrode low-pass filtering effect and the “dip” effect, which strongly modify the signal spectrum [19,33]. Large IED values (IED > 10 mm) are often preferred because they detect sources from a larger volume and provide larger signals. However, they are more sensitive (a) to crosstalk from neighboring muscles and (b) to the effect of MUAP generation at the IZ and MUAP extinction at the muscle–tendon junction (EOF effect). In addition, for the reasons presented above, large IED values (IED > 10 mm) are not suitable for estimating muscle fiber CV, for which at least four electrodes on the same side of the IZ are usually required as well as the most suitable DD derivation. Finally, the features of different sEMG signals cannot be compared when the signals are collected with electrodes of different sizes, distances, and locations with respect to the IZ(s). The effects of these factors on the amplitude and spectral sEMG features of the upper trapezius and knee extensors are clearly explained in [37,38].

The above considerations apply in general and also specifically in the case of muscles that are located very close to the skin surface with thin skin and subcutaneous tissue. In the case of more distantly located muscles or subjects with a higher body mass index, these considerations may be somewhat relaxed because of the low-pass filtering effect introduced by the increased distance between the source and detection points. This factor reduces the bandwidth of the sEMG signal in space and time. This issue has been investigated by Minetto et al. [30], who concluded that because of the relevance of non-propagating components (due to the EOF effect and crosstalk), muscle fiber CV (when of interest) cannot be reliably estimated in these cases: “Interelectrode distance greater than 10 mm is not only recommended but required in sEMG detection from the vastus medialis and lateralis (VM and VL) of obese subjects to counteract the sEMG amplitude attenuation due to the increased thickness of the subcutaneous layer.” Since, in these cases, the sEMG power spectrum is narrower and the shortest wavelength in space is longer, it can then be concluded that IEDs of 5–10 mm should be used to investigate the superficial muscles of lean subjects while IEDs of 10–20 mm could be used in studies concerning muscles that are located more distantly, especially in subjects with a greater body mass index.

Similar considerations apply to the issue of electrode size. The option to select IED implies adjustable selectivity, which is not possible with coaxial needles. The issue of electrode location, size, and IED becomes particularly important when electrode grids are used, spatial interpolation is applied or individual MUAPs are to be analyzed.

## 4. Factors That Can Affect sEMG and Its Physiological Interpretation

The distribution of sEMG potential is a time-evolving monopolar image (a movie) whose full information content can only be recovered and used when the image is properly sampled in space and time. However, most current clinical applications are based on traditional bipolar (SD) readings since the obtained information is, in general, appropriate for answering clinical questions with an adequate compromise between the in-field feasibility of the activity detection of many muscles and the reduction in information on the active MUs.

It is important to understand that a bipolar recording is nothing more than taking two samples in space out of the monopolar map and plotting their instantaneous voltage difference versus time. When using bipolar detection, some of the strict requirements concerning the electrode size and IED of an electrode grid can be somewhat relaxed in favor of a larger detection volume and lower noise, at the price of lower selectivity and a greater risk of crosstalk. Electrodes that are located improperly or have large sizes and distances cause waveform changes (Figure 18, Figure 19 and Figure 20) and an often-undetected sensitivity to crosstalk, as shown for the brachioradialis muscle by Merlo et al. in Special Issue [39].

In addition, during dynamic contractions, the muscle moves under the electrodes and this may cause changes in the sEMG amplitude that are only due to the changes in the relative IZ position. These issues are rarely discussed in the clinical literature or addressed in schools or courses and sEMG users seem not to be aware of them. Consequently, incorrect conclusions may be reached by unaware users.

### 4.1. Skin Treatment

Proper skin treatment is important for both bipolar and HDsEMG recordings. In HDsEMG, the relatively large number of electrodes increases the likelihood of bad contact and requires the accurate treatment of the skin (e.g., shaving, rubbing with peeling cream, and then cleaning with a damp cloth), preferably without using alcohol, which leaves skin dry [31]. This requires some training in skin treatment and proper grid application, with uniform pressure on each electrode and no wrinkles. When conductive paste or gel is applied to arrays or pairs of electrodes, gel bridges between the electrodes must be avoided. The performance and repeatability of results rely on proper electrode choice, placement, and management, keeping in mind that small electrodes are noisier and more selective than large electrodes but introduce less signal filtering, as discussed above and indicated in Figure 18 and Figure 19. It is also important to point out that the two electrode–skin impedances of an electrode pair must be small and similar to reduce power line interference [20].

### 4.2. Movement of the Muscle under the Electrodes

**Figure 21:**
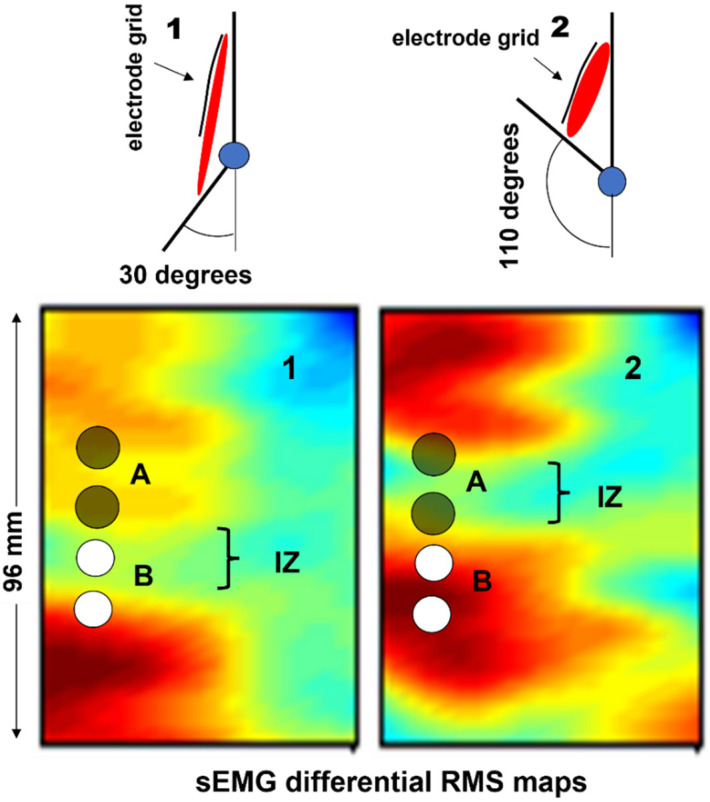
Example of a biceps brachii muscle shortening under the skin during a dynamic contraction. When the forearm flexes from 150° to 70° (internal angle with respect to the arm), the biceps muscle shortens under the array and the map changes from Map 1 to Map 2. Considering the white and the black pair of electrodes as being positioned by operators A and B, the white pair would indicate that elbow flexion causes an increase in the sEMG amplitude whereas the black pair would indicate the opposite. Great caution must be used during the placement of single electrode pairs for the study of dynamic contractions because the muscle geometry changes taking place under the electrodes cause sEMG variations that may be erroneously attributed to changes in the neural muscle drive when electrodes are placed on or near to the innervation zones (IZs). Adapted with permission from Figure 6.5 of Ref. [22]. Copyright Springer Verlag Italia, 2012.

Figure 21 addresses the important problem of muscles sliding under the skin during dynamic contractions. The example shows two interpolated images obtained using an electrode grid that is placed on a biceps brachii. When the forearm flexes from 150° to 70° (internal angle between arm and forearm), the biceps muscle shortens under the array and the EMG amplitude map changes from Map 1 to Map 2. Considering one operator placing the white pair of electrodes and another operator placing the black pair, the first would conclude that elbow flexion increases the sEMG amplitude but the second would conclude the opposite. The change in sEMG amplitude that is detected by the two electrode pairs when the elbow flexes is due to the change in geometry, not the change in muscle electrical activity. This is an important issue to consider during dynamic contractions and is likely to be one of the reasons for the lack of uniform results reported in the literature, in addition to electrode size and IED. Users should be aware of this phenomenon, which takes place in long fusiform muscle-spanning joints with large angle variations. Although only examples from biceps brachii have been reported, other muscles may show the same phenomenon. An atlas for proper electrode placement should always be used when dealing with bipolar sEMG. It should also be verified that the electrode pair remains on the same side of the muscle IZ(s) during the entire range of movement. Since this is impossible when using bipolar electrodes, atlases that indicate the regions where electrodes should not be placed have been made available [22].

### 4.3. Identification of Innervation Zones

The identification of the IZ by means of linear electrode arrays or HDsEMG, as reported in Section 2.3, has more potential clinical applications other than being useful for supporting the proper positioning (or selection) of a pair of electrodes [40] (far from IZs and on minimum crosstalk areas), including the targeted injection of the botulinum neurotoxin, which is commonly used to treat disorders such as cerebral palsy, spasticity, and cervical dystonia [4,5,41].

When the toxin is applied to the IZ, a lower dose can be highly effective [4,5,42]. The use of HDsEMG to target the IZ could reduce the amount of injected toxin and, consequently, limit the treatment cost [43]. An atlas of muscle innervation zones is available for most superficial muscles [22].

**Figure 22:**
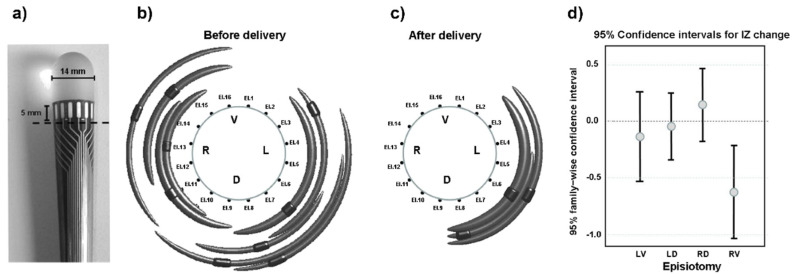
A study of the anal sphincter pre- and post-episiotomy: (**a**) the anal probe with 16 silver electrodes equi-spaced along the circumference; the detection of the innervation zones (IZs) in the same woman before (**b**) and after (**c**) delivery with episiotomy. The black cylinders represent the neuromuscular junctions and the gray arcs depict the identified motor units (MUs). In this subject, no MUs were detected on the side where the episiotomy was performed. The results from 82 cases of episiotomy are shown in (**d**). Fewer MUs were detected on the right ventral (RV) quadrant, where the episiotomies were performed, suggesting damage to the innervation following surgery. Note: the information collected before the delivery was not used to plan the surgery. Quadrants: LV, left ventral; LD, left dorsal; RD, right dorsal; RV, right ventral. Adapted with permission from Figures 1, 2, and 4 from Ref. [45]. Copyright The International Urogynecological Association, 2014.

The identification of the IZ is also a useful tool for planning surgery, for example, in the case of episiotomy during child delivery. The innervation to the external anal sphincter muscle may be damaged due to episiotomy, thereby increasing the chances of future fecal incontinence [44]. The European project OASIS and the multicenter study TASI exploited multichannel sEMG probes to identify changes in the external anal sphincter innervation in women who underwent episiotomy during labor. It was found that (a) the distribution of IZs is highly variable across individuals [45] but can be identified in a repeatable way across days [46] and (b) the number of IZs significantly decreases in the quadrant where the episiotomy was performed [45]. Therefore, the identification of the IZs prior to delivery could be a preventive measure to guide the episiotomy incision (when necessary) and to suggest the ways or sides with the least risk. Figure 22 depicts the probe that was used, one significant result, and the statistical results from 82 cases of episiotomy.

Despite the literature in this field [45,46,47], the clinical use of sEMG is still absent because of the lack of information and teaching for obstetricians, midwives, and pelvic floor therapists.

### 4.4. Examples of HDsEMG Applications in Neurophysiology

A comprehensive overview of all of the clinically possible applications of single channel and HDsEMG in neurology exceeds the purpose of this paper. Therefore, only a few relevant examples are highlighted in the following section to underline the need for training with a view to future applications. For a more comprehensive analysis, refer to the systematic reviews in [7,9,32].

HDsEMG was already being used in the early 1990s to differentiate the pathologies of neuronal origin from those of muscular origin. Ramerkers et al. [48] were able to show that when using HDsEMG with the NDD detection system (Figure 16), the resulting signals that are derived from patients with muscular or neuronal disorders exhibit disease-specific features.

**Figure 23:**
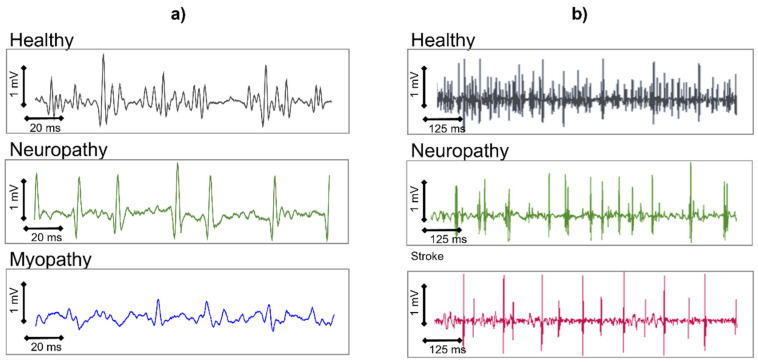
Characteristic changes in the normal double differential spatially filtered (Figure 16) high-density sEMG (HDsEMG) signal associated with different disorders: (**a**) myopathies lead to a change in the shape of the motor unit action potentials (MUAPs) while neuropathies lead to a decrease in the number of MUAPs contributing to the signal; (**b**) neuromuscular disorders involving the upper motor neuron, such as stroke, are characterized in the spatially filtered HDsEMG signal not only by fewer active motor units, but also by a lower variability in firing rate [49,54]. For a better representation of these effects, (**a**,**b**) have different temporal scales.

Figure 23a shows the NDD spatially filtered signal as it manifests in neuropathies or myopathies. While myopathies are mainly characterized by a change in the shape of the MUAPs, neuropathies show a decrease in the detected number of active MUs. Simulation studies have indicated that in the case of myopathies, the degeneration of muscle fibers is responsible for the changes in the signal, whereas a loss of Mus leads to the characteristic changes found in neuropathies [26]. These typical changes in the spatially filtered HDsEMG signal that characterize muscular or neuronal pathologies can be described by seven parameters and enable correct diagnostic conclusions with an average accuracy of 97% [49]. This means that the possible diagnostic yield of the non-invasive HDsEMG technique, for superficial muscles, is in the range of needle electromyography [50,51]. Other applications refer to the MU number estimation, the quantification of fasciculations [52], HDsEMG changes in the muscle structure, and MU recruitment of stroke patients in pathophysiology studies [53].

As HDsEMG is a non-invasive method, it is particularly useful for monitoring the progress of neuromuscular disorders in children. Furthermore, for superficial muscles, HDsEMG provides additional information about the MUs and their recruitment, which is not accessible through needle EMG but could be relevant for diagnostic purposes. This includes information about the CVs of MUAPs along the muscle fibers, as well as the spatial distribution of excitation within a muscle.

**Figure 24:**
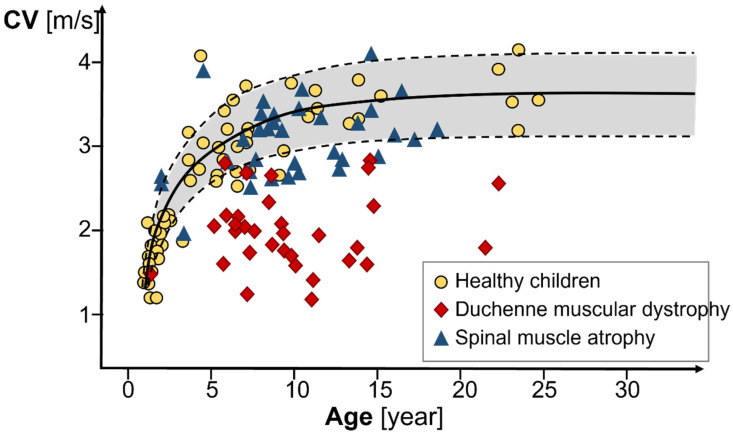
Change in the mean conduction velocities of motor unit action potentials (MUAP CVs) with age in healthy subjects and in patients suffering from Duchenne muscular dystrophy or spinal muscle atrophy. The solid black line is the regression curve for the healthy subjects. The gray area contains 95% of all healthy subjects. Adapted with permission from Ref. [55]. Copyright Muscle and Nerve, 1997.

Figure 24 indicates how the MUAP CV changes as children develop. The most significant age-related change in MUAP CV occurs at an age of up to 5 years. From an approximate age of 15 years, the MUAP CV remains constant. The CVs of the MUAPs are significantly reduced in patients with Duchenne muscular dystrophy, compared to healthy children [55], and in patients suffering from carpal tunnel syndrome [56].

Many other neurological applications of HDsEMG are clinically relevant. Among these are hypokalemic periodic paralysis, pathological fatigue, and MU size estimation, presented in the reviews by Zwarts et al. [7], Drost et al. [9], and Hogrel [32], along with a discussion of the advantages and limitations of HDsEMG.

Numerous applications of HDsEMG are being developed in research labs to decode the neural drive to muscles by means of the decomposition of the sEMG into the constituent MUAP trains. These methods are based on the early work of Holobar and Zazula [23] and are ready for clinical evaluation.

Finally, it is fair to say that medical devices can only be used within their “intended use” (what can and cannot be carried out with that device), which is indicated by the producer in the first pages of their user manual. Any diagnostic tool requires a class II medical device certification in Europe, an FDA approval in the USA or a similar certification in other countries. Users and researcher should verify the certification level before buying or using sEMG devices for specific applications.

### 4.5. Issues of Bipolar sEMG Detection in Neurorehabilitation Applications

In neurorehabilitation, the use of motion analysis with sEMG clearly influences treatment planning in multiple patient populations and can lead to better outcomes when properly used [57]. Gait analysis is a functional evaluation that provides a quantification of the observed performance and identifies motor deficits and the underlying causes of the observed pathological patterns [58,59,60]. This evaluation allows for the formulation of a precise and targeted therapeutic plan, such as surgical, pharmacological, orthotics or physiotherapy plans [57,58,61,62,63,64]. In the study of muscle activation during the gait of neurological patients, it is of paramount importance to avoid common errors that could possibly lead to incorrect clinical decisions [39,65,66]. The literature provides guidance on how to properly perform an examination so that it can be reliable and used for functional assessments and clinical evaluations [67].

**Figure 25:**
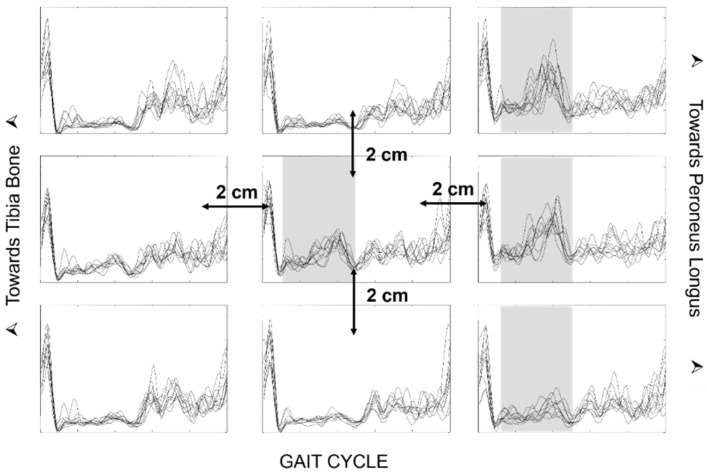
Envelopes of bipolar sEMG (one plot per stride for 10 strides) from nine electrode pairs obtained by placing a 4 × 3 grid of electrodes, 15 × 18 mm in size, over the anterior aspect of the leg. The medial column of electrodes is placed on the edge between the tibialis anterior muscle and the tibia bone. The gray bands indicate the presence of activity in the mid and terminal stances due to signals coming from the peroneus longus. Adapted with permission from Ref. [66]. Copyright Journ. of Electromyography and kinesiology, 2007.

**Figure 26:**
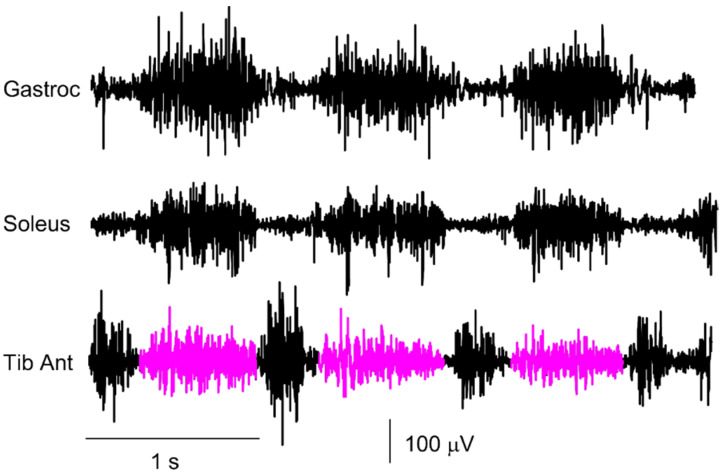
Example of sEMG signals acquired during gait analysis of the leg of a healthy 10-year-old boy by means of pairs of circular electrodes: diameter of the conductive region = 10 mm; detection area = 78.5 mm^2^; center to center distance = 20 mm. Crosstalk on the tibialis anterior from the plantar flexors is outlined in purple. Reproduced from Ref. [72].

The main errors in the use of sEMG for the evaluation of neurological patients are attributable to the type of electrodes, the placement protocol, and the presentation of the data that are chosen. The traditionally used SENIAM recommendations for electrode placement [68] may not consider the problem of crosstalk, as indicated in Figure 25 and Figure 26. In order for the evaluation to be clinically usable, it is necessary for the chosen electrode placement protocol to guarantee the selectivity of the analyzed muscle activation, with small electrodes placed on the so-called minimum crosstalk areas [39,69,70,71] and on the same side as (and far away from) the IZs [22].

The width of the electrodes and the IED can also play a crucial role, especially when recording the activity of small muscles, such those as in children. The disposable ECG pre-gelled electrodes that are normally used in clinical evaluations are often too large. When the electrode contact area is wider than the target muscle, it can cover more than one muscle, thus reporting an incorrect activation time due to crosstalk.

Finally, it is important that the reports of the examinations carried out contain raw traces to distinguish the sEMG signal from other possible artifacts [73] that are very frequent in the study of neurological patients with movement alterations. In fact, the representation of the sEMG signal in the gait cycle through the envelope, although more synthetic, can be dangerous and misleading. It is important to always visually verify the quality of the raw sEMG signals since after the computation of the amplitude envelope, artifacts and interferences are no longer detectable and cannot be eliminated [74].

**Figure 27:**
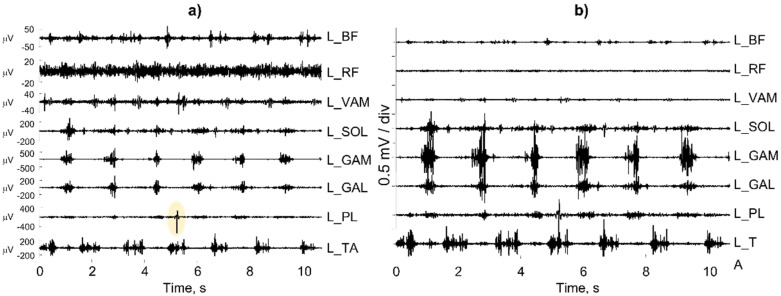
Bipolar sEMG recordings from eight muscles of the left leg during the gait analysis of a subject: (**a**) “autoscale on”; (**b**) same scale for all channels. A superficial visual analysis of (**a**) would lead to incorrect conclusions.

The issue of proper signal representation and scaling (of the electrodes and not the output of the amplifier) may seem obvious and trivial, but it is not [73]. Figure 27a shows a number of individual sEMG bipolar channels recorded from muscles in the left leg of a subject with a continuous activity of the rectus femoris (L_RF). A more careful analysis reveals that the operator selected the “autoscale” option of the software instead of the “fixed scale” option. Figure 27b shows the same channels with the same scale (0.5 mV/div for the input signal), but the picture is very different. In addition, the “autoscale” option automatically selects the scale of the L_PL muscle on the basis of the artifact outlined in yellow.

### 4.6. Issues in Ergonomics, Occupational Medicine, and Sport Sciences Applications

Surface EMG is fundamental for the proper design of workstations and chairs, the planning of sport training, and the understanding and prevention of neck, shoulder, and back pain [15,75,76,77].

Ergonomics, occupational medicine, and sport sciences require specific competencies and training in sEMG. These areas are affected by the barriers and difficulties that have been discussed elsewhere [78]. The early NIOSH publication by Soderberg [79], the book by Kumar and Mital [80], the review by Marras [81], and the recent works of Gazzoni et al. [82] and Varecchia et al. [83], among many other contributions, underline the importance of teaching the proper use of sEMG in ergonomics and occupational medicine. Similarly, the early work of Clarys [84], Chapter 9 of *Electrodiagnosis in New Frontiers of Clinical Research* [85], and the recent work of Felici [77], among many other works, outline 30 years of efforts in the field of sEMG use in sports and exercise. Following this long history of research, “There is a critical need to … instruct teachers across the disciplines of these fundamentals… and give access of these developments to the current generation of students, which would improve the translation in order to potentially predict and cure pathologies of the neuromuscular function. This has an impact, for example, on the student awareness level of some very simple points related to the correct collection of sEMG data. Issues such as electrode location, IZ detection, skin preparation, movement artifacts and sweating are very often ignored.” [77].

In addition, Martin and Acosta-Sojo [78] indicated that “The application of sEMG requires knowledge (e.g., EMG theory, signal processing, and neurophysiology), experience (e.g., electrode placement and signal morphology), and time (e.g., experimental design and procedure). These essential components may be deepened in doctoral studies but are not provided in clinical curricula such as physiotherapy and occupational therapy. Hence, broadening or amending the educational programs of physiotherapists, occupational therapists, movement scientists, and ergonomists (as future clinical users of sEMG) should prove useful.”

Further considerations for sEMG applications in sports and ergonomics are relevant and discussed in the extensive literature [83,86] but exceed the purpose of this work. In all studies, the proper acquisition of sEMG is fundamental to reaching consistent conclusions.

The published literature [19,20,36,87] contains summary tables of all of the main factors that may affect correct signal estimation and the basic guidelines to follow for proper EMG recording.

## 5. Discussion and Limitations of the Work

As indicated in the introduction, this work does not present innovative research results concerning sEMG hardware, processing techniques or applications. Rather, it presents (a) a number of the “first level” fundamental concepts of sEMG detection and interpretation that clinical operators should be familiar with and (b) some model-based teaching tools that could be useful to teach these concepts. All of these concepts can be found in textbooks and tutorials, in which they are presented within the mathematical framework that engineers are familiar with. This framework is unacceptable to most clinical operators, who have neither the need nor the background required to understand it. This work provides material and suggestions concerning the teaching of these concepts and their transfer into the educational curricula of clinical figures.

The same applies to other “second level” technical concepts, such as the spectral analysis of sEMG, the myoelectric manifestations of muscle fatigue, image segmentation and the definition of regions of muscle activity, the decomposition of interferential EMG into the constituent MUAP trains, the association between sEMG and biomechanical variables, sEMG in dynamic conditions, etc. These concepts are not discussed in this work, which is focused mostly on the fundamental issues of sEMG detection and simple model-based interpretation and teaching.

Surface EMG is very rich in information that can be extracted to draw conclusions about the coordination between muscles and muscle groups [88] and the anatomy, physiology, and pathology of a muscle and to monitor muscle changes following pathologies, training or therapeutic interventions. The proper understanding of sEMG generation and detection processes, as well as the visual understanding of the raw signals and the verification of good quality, is a fundamental preliminary step for the subsequent computer-assisted extraction of features (e.g., muscle fiber CV, amplitude and spectral variables, etc.) of sEMG to avoid the GIGO (garbage in, garbage out) effect.

A number of textbooks, recommendations, tutorials, consensus papers, and editorial projects have been published concerning these issues [17,19,20,22,34,35,36,89,90]. They include indications of the procedures to follow in order to (a) allow other researchers to reproduce the published work, (b) indicate whether or not results may be compared to those of others, and (c) promote the preparation of standards that account for clinical experiences. Despite the extensive literature concerning these recommendations, the published articles discuss a great variety of sEMG electrode sizes, distances, and locations. Sometimes, this information is not even reported or incorrectly reported, as indicated in the recent review by Merlo et al. in Special Issue [39]. A greater awareness of the problem is required by reviewers, journals, and schools, as well as by scientific and professional societies within the field.

A fundamental issue is the technology transfer from research labs to professional and medical schools, as well as to healthcare institutions. The simplification of concepts (as is often demanded by clinical operators and attempted here) and the availability of free teaching materials and recommendations are by far insufficient. As Rogers [91] notes, innovations are initially perceived as uncertain and even potentially negative. To overcome this uncertainty, most people seek out colleagues who have already adopted the new idea. Thus, the diffusion process is initiated by a few individuals who see the advantages in adopting an innovation and then spread the word among their colleagues or acquaintances, which is a process that takes years, regardless of the availability of textbooks, recommendations, publications, and teaching materials (which is often disregarded) [92]. Every change requires intellectual and learning efforts by the user and the view of such effort as a social, as well as economical, process [86,88,93] (see the EMG Reporting Standards of the International Society of Electrophysiology and Kinesiology, available online: https://isek.org/emg-standards, https://www.tudelft.nl/en/education/programmes/bachelors/kt/bachelor-of-clinical-technology/, and https://www.tudelft.nl/onderwijs/opleidingen/masters/technical-medicine/msc-technical-medicine/).

As in the case of other bioelectric signals, it is up to the clinical operator to promote and use newly available technical information that is suitable for properly answering clinical questions. Learning about this in school is important; however, the fields of rehabilitation technology and neuromuscular instrumentation are becoming so extensive that it is hard to fit even the basic concepts discussed in this work into the few academic years that lead to a degree in physiotherapy, sport and movement sciences or ergonomics. To some extent, this is true in other healthcare fields as well and has justified the training of new interdisciplinary professional health figures, such as the “clinical technologist” that trains at the Technical University of Delft (NL) (https://www.tudelft.nl/en/education/programmes/bachelors/kt/bachelor-of-clinical-technology/, and https://www.tudelft.nl/onderwijs/opleidingen/masters/technical-medicine/msc-technical-medicine/) within the BS and MS curricula for courses on “advanced signal acquisition”, “sensing neurophysiological signals”, and the “electrostimulation of the neurophysiological system”. These new figures may solve the problem of the lack of time to teach measurement techniques, such as sEMG, to strictly clinical operators. This issue of the lack of time has been pointed out repeatedly in a number of surveys [17,94]. These new interdisciplinary figures may help to address problems that can be solved by technology that are not yet perceived as important by clinicians.

## 6. Conclusions

Surface EMG technology is undergoing very fast development, which allows for the non-invasive investigation of movement, muscle function, and nervous system control strategies. This progress is much faster than the process of knowledge transfer, meaning that the knowledge gap is widening [3,32,92,95,96]. The correction of this situation requires the broadening of educational programs and the competence of the clinical users of sEMG, such as physiotherapists, occupational therapists, movement scientists, ergonomists, physical medicine and rehabilitation physicians, and clinical neurologists. This work indicates some options, tutorials, and tools that are available for motivating and training teachers, students, and clinicians for the challenges that they may face within the sEMG field. Existing tools and tutorials are mostly open-access and others are in preparation.

Many more materials should be developed, based on the experience of teachers and clinicians in the field, to provide explanations and descriptions of sEMG features (not discussed in this work) using analogies and user-friendly models.

The success of any future hardware/software technology to extract information from sEMG fundamentally depends on the ability of the operator to collect “good” sEMG signals from the desired muscles that are free from artifacts and power line interference and have minimal crosstalk from other muscles. The proper training and academic education of operators is a necessary condition for the success of the available, as well as future, technologies within this field.

## Data Availability

Not applicable.

## Supplementary Materials

The following supporting information can be downloaded at: https://www.mdpi.com/article/10.3390/s22114150/s1, Figure 2: Sup1, Figure 4: Sup2, Figure 5: Sup3, Figure 7: Sup4, Figure 11: Sup5, Figure 12: Sup6, Figure 13: Sup7, Figure 14: Sup8, Figure 15: Sup9, Figure 18: Sup10, Figure 20: Sup11.

## Acronyms

EEG	Electroencephalogram
ECG	Electrocardiogram
EMG	Electromyogram/electromyography
sEMG	Surface electromyogram
HDsEMG	High-density sEMG
IZ	Innervation zone of a motor unit or muscle
MU	Motor unit
MUAP	Motor unit action potential
NMJ	Neuromuscular junction
IED	Interelectrode distance
EOF	End-of-fiber
CV	Conduction velocity of muscle fibers
RMS	Root mean square
SD	Single differential (bipolar)
DD	Double differential
NDD	Normal DD or Laplacian
VL	Vastus lateralis
VM	Vastus medialis
