# Supplementary material for: Fundamental Concepts of Bipolar and High-Density Surface EMG Understanding and Teaching for Clinical, Occupational, and Sport Applications: Origin, Detection, and Main Errors
Source: Sensors (Basel). 2022 May 30;22(11):4150. doi: 10.3390/s22114150 (PMC9185290; doi:10.3390/s22114150)
Supplement: Supplementary file 1 [file sensors-22-04150-s001.zip › Sup1_Figure_2.pptx]

## Slide 1
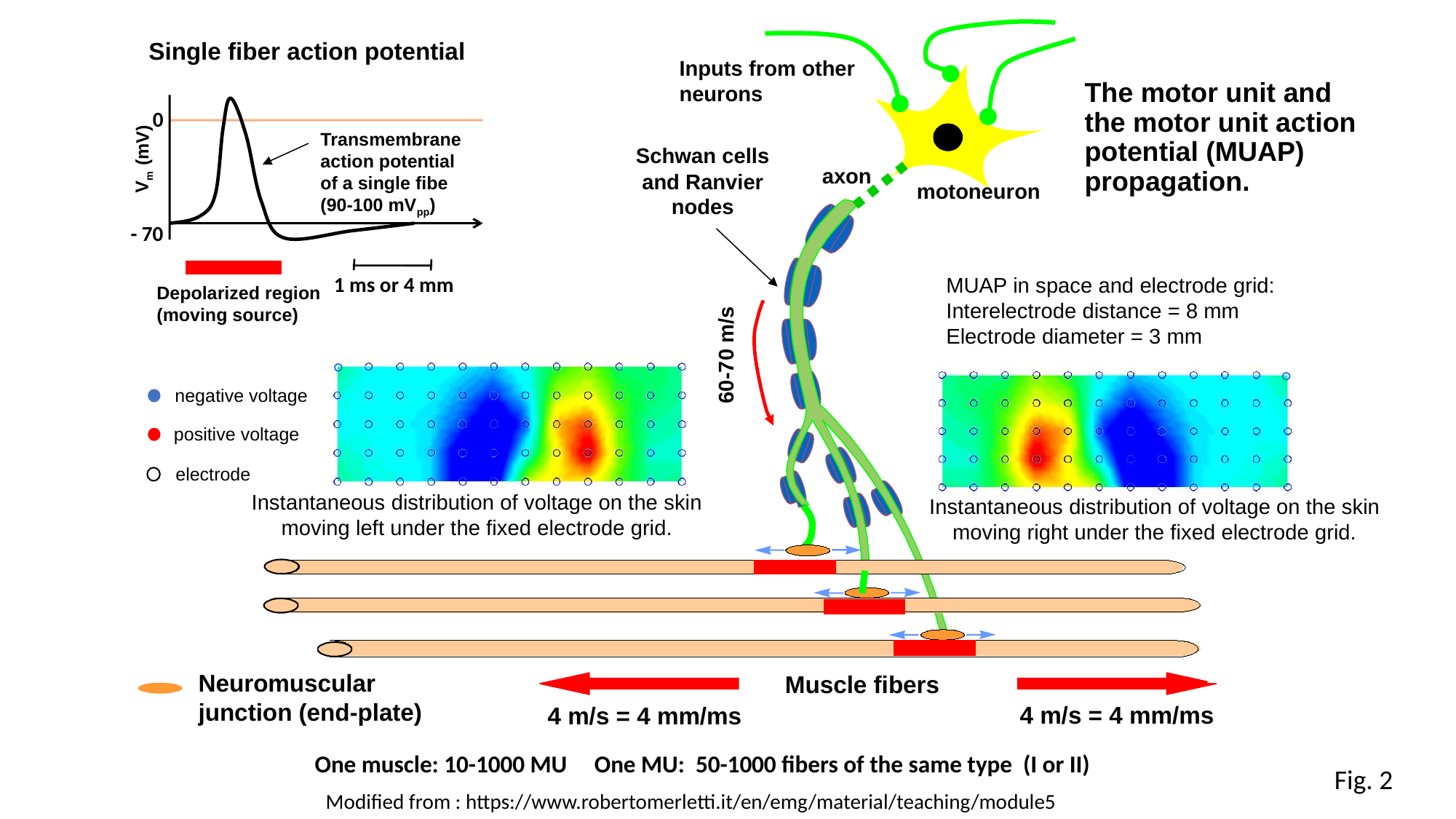

Single fiber action potential
Inputs from other neurons
The motor unit and
the motor unit action potential (MUAP) propagation.
0
Transmembrane action potential of a single fibe
(90-100 mVpp)
Vm (mV)
- 70
1 ms or 4 mm
Depolarized region
(moving source)
Schwan cells and Ranvier nodes
axon
 motoneuron
MUAP in space and electrode grid:
Interelectrode distance = 8 mm
Electrode diameter = 3 mm
Instantaneous distribution of voltage on the skin moving right under the fixed electrode grid.
60-70 m/s
negative voltage
positive voltage
electrode
Instantaneous distribution of voltage on the skin moving left under the fixed electrode grid.
Neuromuscular junction (end-plate)
Muscle fibers
4 m/s = 4 mm/ms
4 m/s = 4 mm/ms
One muscle: 10-1000 MU One MU: 50-1000 fibers of the same type (I or II)
Fig. 2
Modified from : https://www.robertomerletti.it/en/emg/material/teaching/module5
