# Supplementary material for: Fundamental Concepts of Bipolar and High-Density Surface EMG Understanding and Teaching for Clinical, Occupational, and Sport Applications: Origin, Detection, and Main Errors
Source: Sensors (Basel). 2022 May 30;22(11):4150. doi: 10.3390/s22114150 (PMC9185290; doi:10.3390/s22114150)
Supplement: Supplementary file 1 [file sensors-22-04150-s001.zip › Sup10_Figure_18.pptx]

## Slide 1
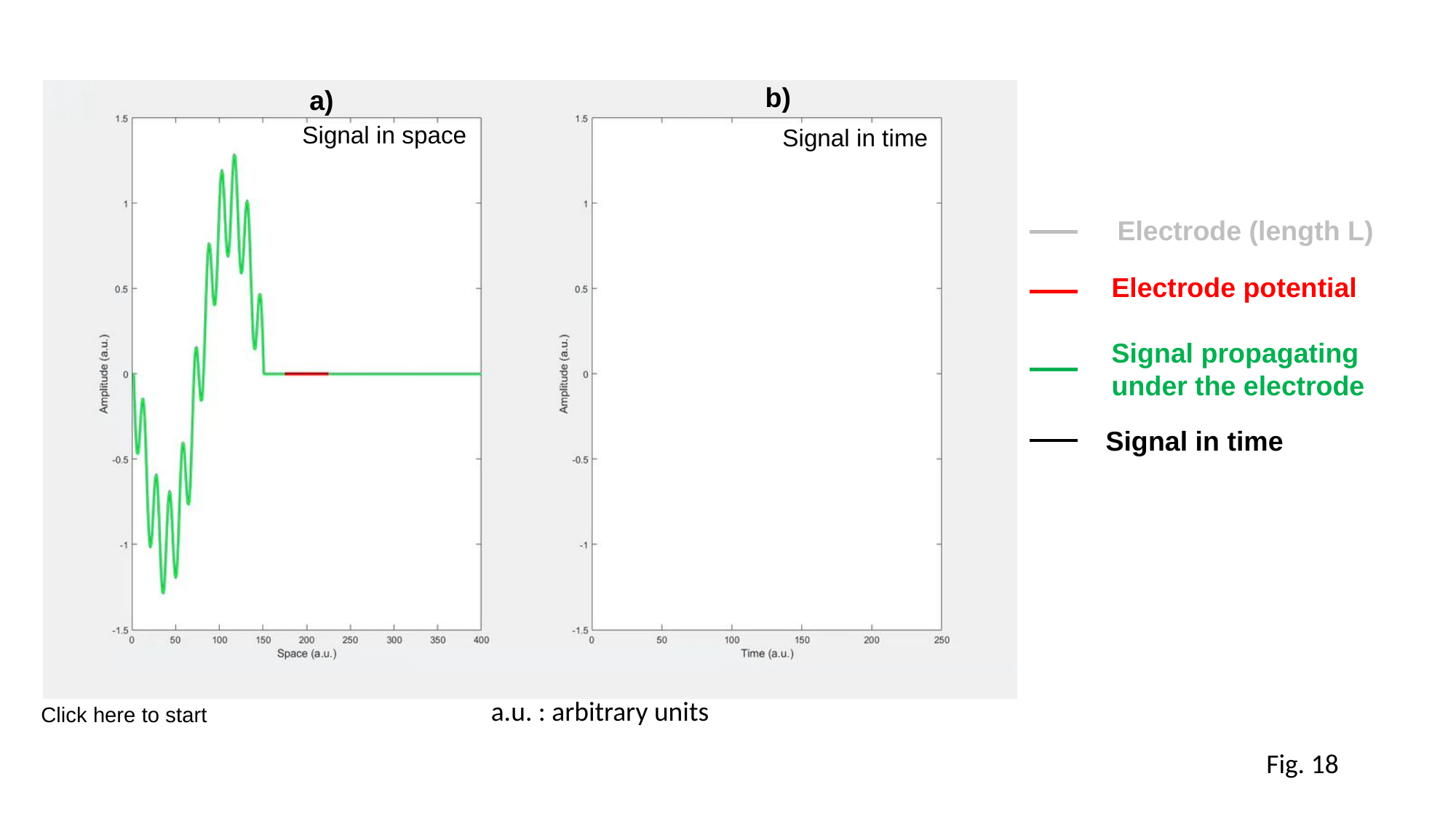

b)
a)
Signal in space
Signal in time
Electrode (length L)
Electrode potential
Signal propagating under the electrode
Signal in time
a.u. : arbitrary units
Click here to start
Fig. 18
