# Supplementary material for: Fundamental Concepts of Bipolar and High-Density Surface EMG Understanding and Teaching for Clinical, Occupational, and Sport Applications: Origin, Detection, and Main Errors
Source: Sensors (Basel). 2022 May 30;22(11):4150. doi: 10.3390/s22114150 (PMC9185290; doi:10.3390/s22114150)
Supplement: Supplementary file 1 [file sensors-22-04150-s001.zip › Sup2_Figure_4.pptx]

## Slide 1
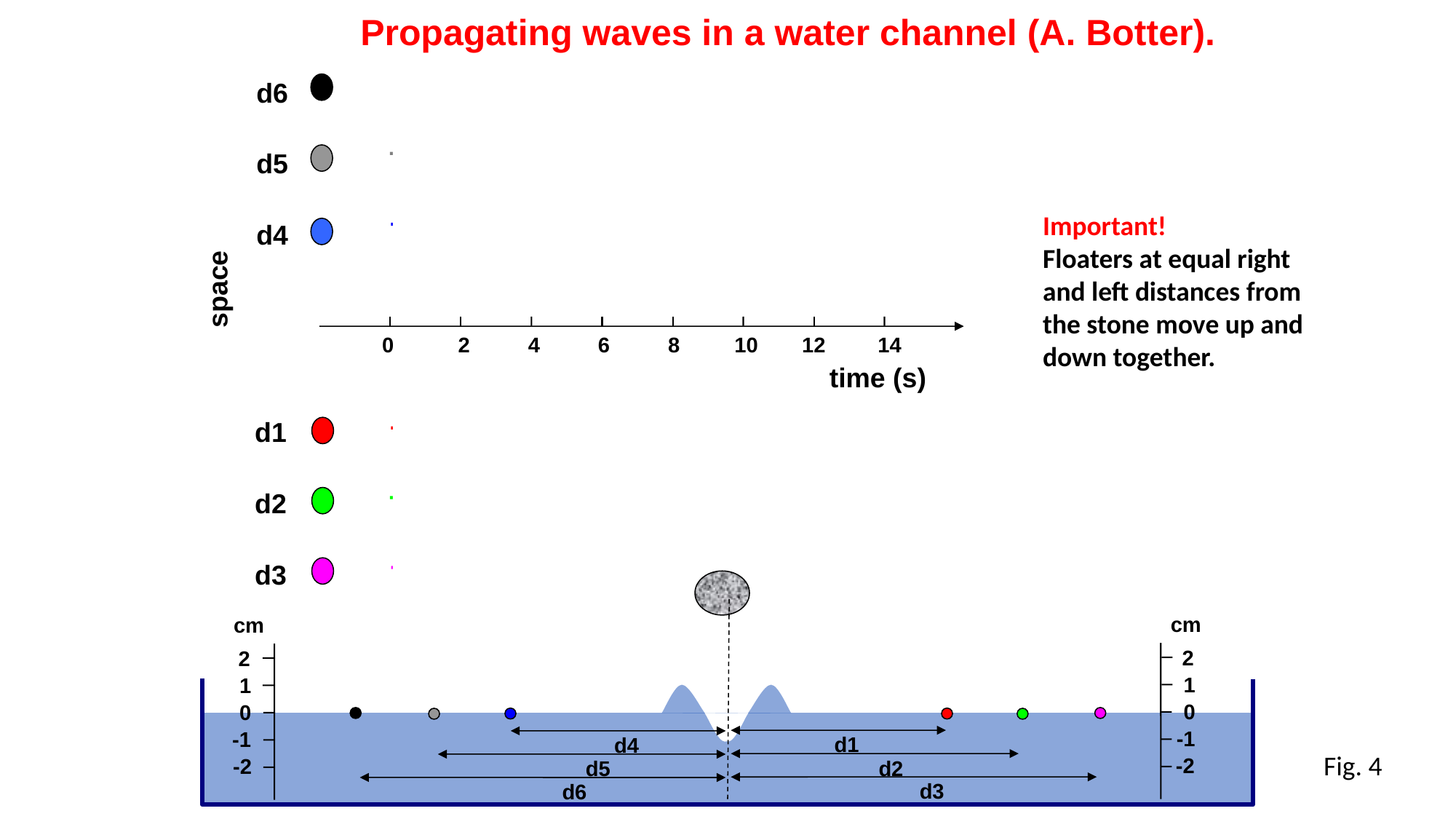

Propagating waves in a water channel (A. Botter).
d6
d5
Important!
Floaters at equal right and left distances from the stone move up and down together.
d4
space
0
 2
 4
 6
 8
 10
12
 14
time (s)
d1
d2
d3
cm
2
1
0
-1
-2
cm
2
1
0
-1
-2
d1
d4
Fig. 4
d2
d5
d3
d6
