# Supplementary material for: Fundamental Concepts of Bipolar and High-Density Surface EMG Understanding and Teaching for Clinical, Occupational, and Sport Applications: Origin, Detection, and Main Errors
Source: Sensors (Basel). 2022 May 30;22(11):4150. doi: 10.3390/s22114150 (PMC9185290; doi:10.3390/s22114150)
Supplement: Supplementary file 1 [file sensors-22-04150-s001.zip › Sup3_FIgure_5.pptx]

## Slide 1
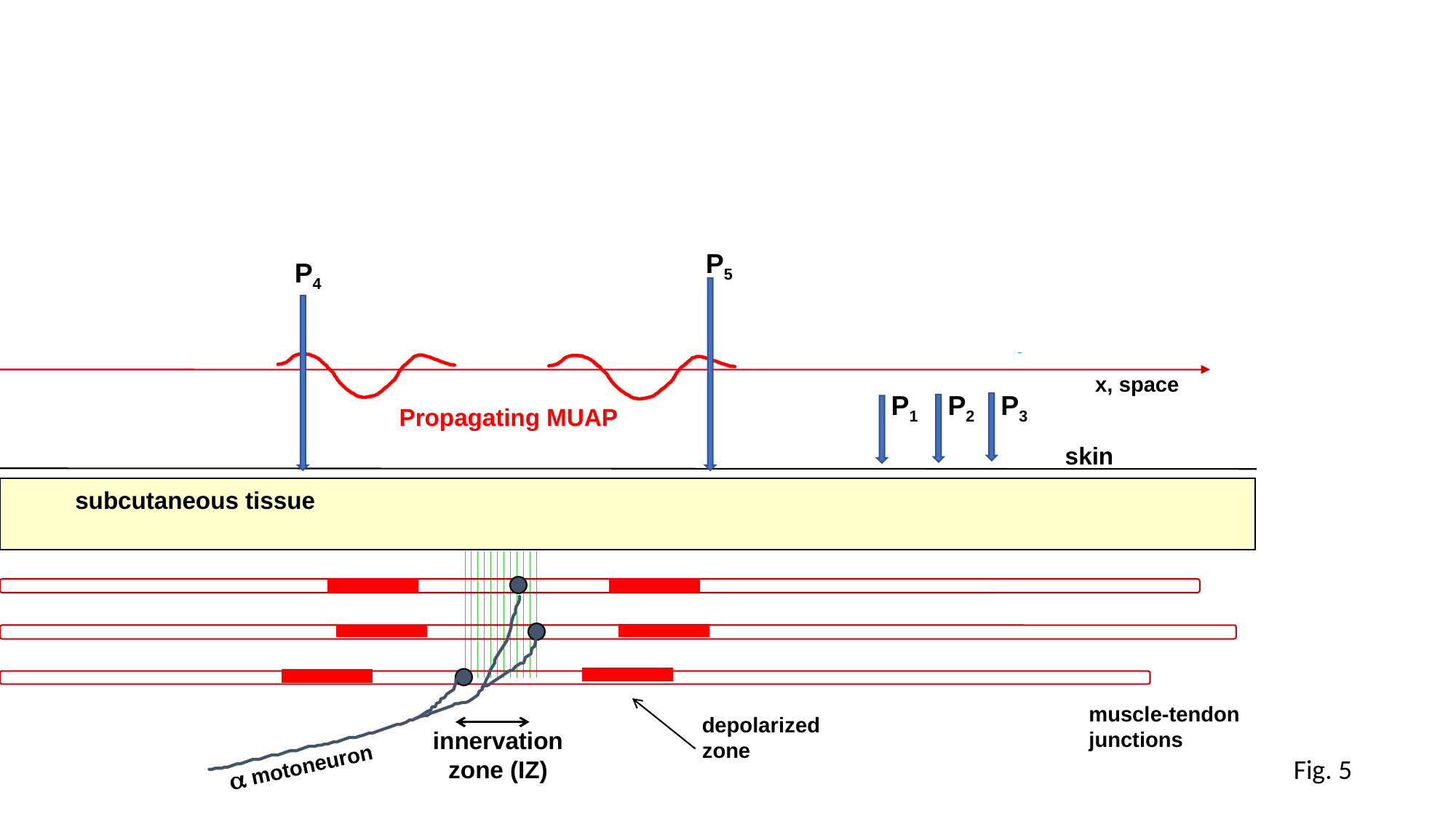

VP1
time
VP(t)
delay
VP2
time
VP3
P5
time
P4
x, space
P1
P2
P3
Propagating MUAP
skin
subcutaneous tissue
muscle-tendon
junctions
depolarized
zone
innervation
zone (IZ)
α motoneuron
Fig. 5
