# Supplementary material for: Fundamental Concepts of Bipolar and High-Density Surface EMG Understanding and Teaching for Clinical, Occupational, and Sport Applications: Origin, Detection, and Main Errors
Source: Sensors (Basel). 2022 May 30;22(11):4150. doi: 10.3390/s22114150 (PMC9185290; doi:10.3390/s22114150)
Supplement: Supplementary file 1 [file sensors-22-04150-s001.zip › Sup4_FIgure_7.pptx]

## Slide 1
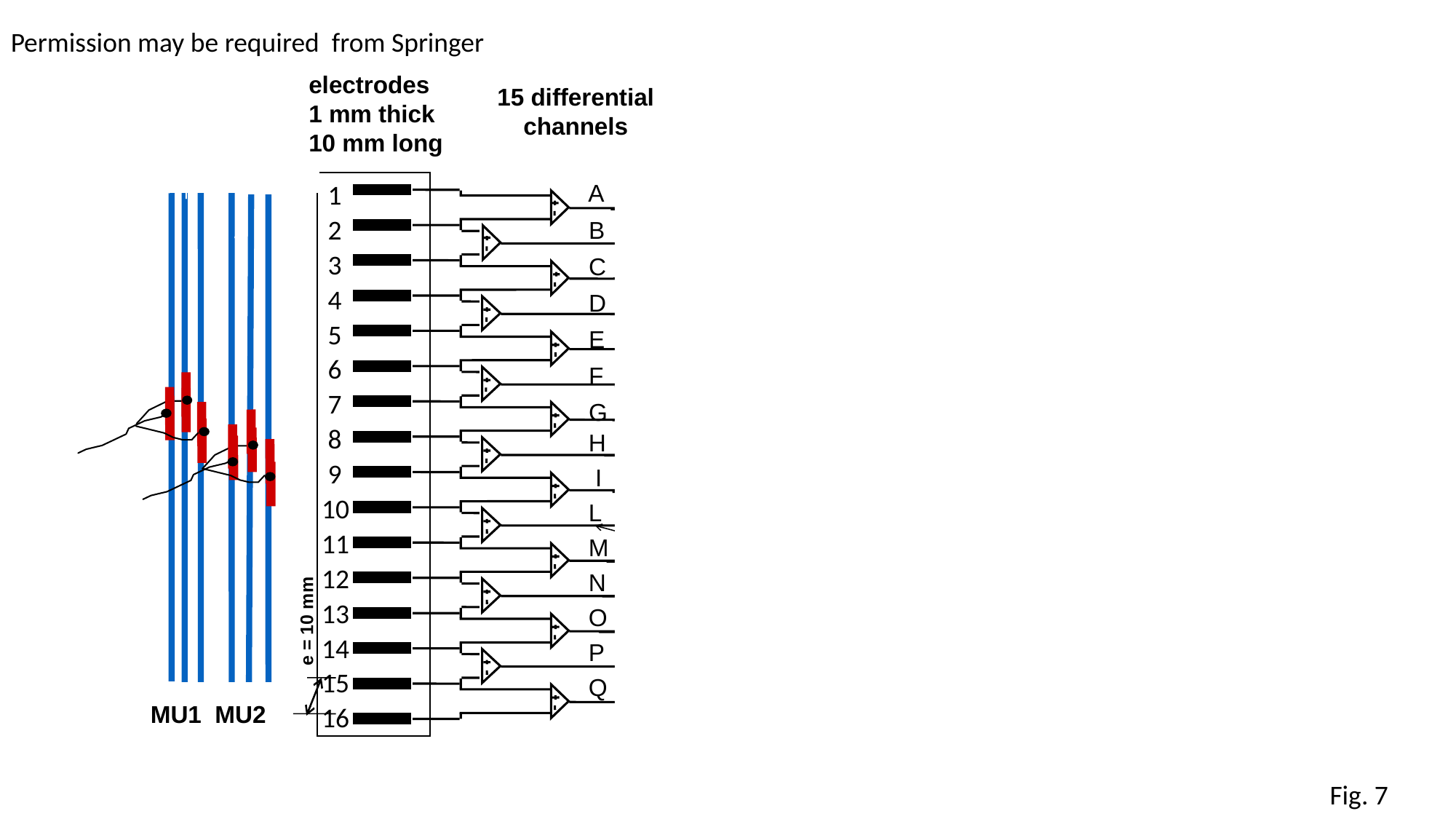

Permission may be required from Springer
electrodes
1 mm thick
10 mm long
15 differential
channels
MUAP1
MUAP2
Differential
Detection
VA = V1-V2
VB = V2-V3
::::
::::
VQ = V15-V16
CV = 40 mm/10 ms = 4 m/s
 A
 B
 C
 D
 E
 F
 G
 1
 2
 3
 4
 5
 6
 7
 8
 9
10
11
12
13
14
15
16
IZ1
H
 I
L
M
N
O
P
Q
time
IZ 2
40mm
10ms
e = 10 mm
MU1 MU2
t2
t1
Fig. 7
