# Supplementary material for: Fundamental Concepts of Bipolar and High-Density Surface EMG Understanding and Teaching for Clinical, Occupational, and Sport Applications: Origin, Detection, and Main Errors
Source: Sensors (Basel). 2022 May 30;22(11):4150. doi: 10.3390/s22114150 (PMC9185290; doi:10.3390/s22114150)
Supplement: Supplementary file 1 [file sensors-22-04150-s001.zip › Sup7_Figure_13.pptx]

## Slide 1
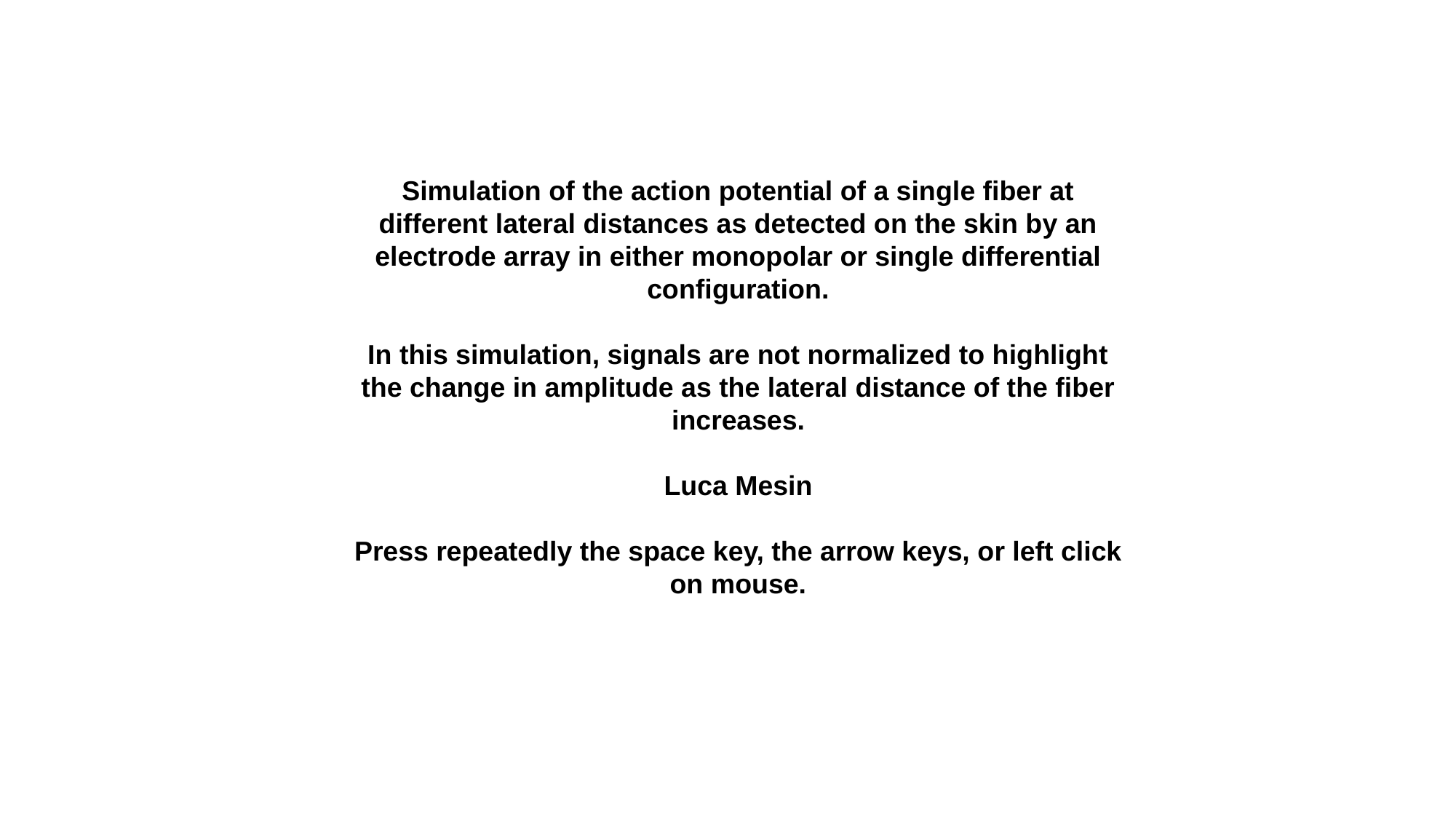

Simulation of the action potential of a single fiber at different lateral distances as detected on the skin by an electrode array in either monopolar or single differential configuration.
In this simulation, signals are not normalized to highlight the change in amplitude as the lateral distance of the fiber increases.
Luca Mesin
Press repeatedly the space key, the arrow keys, or left click on mouse.

## Slide 2
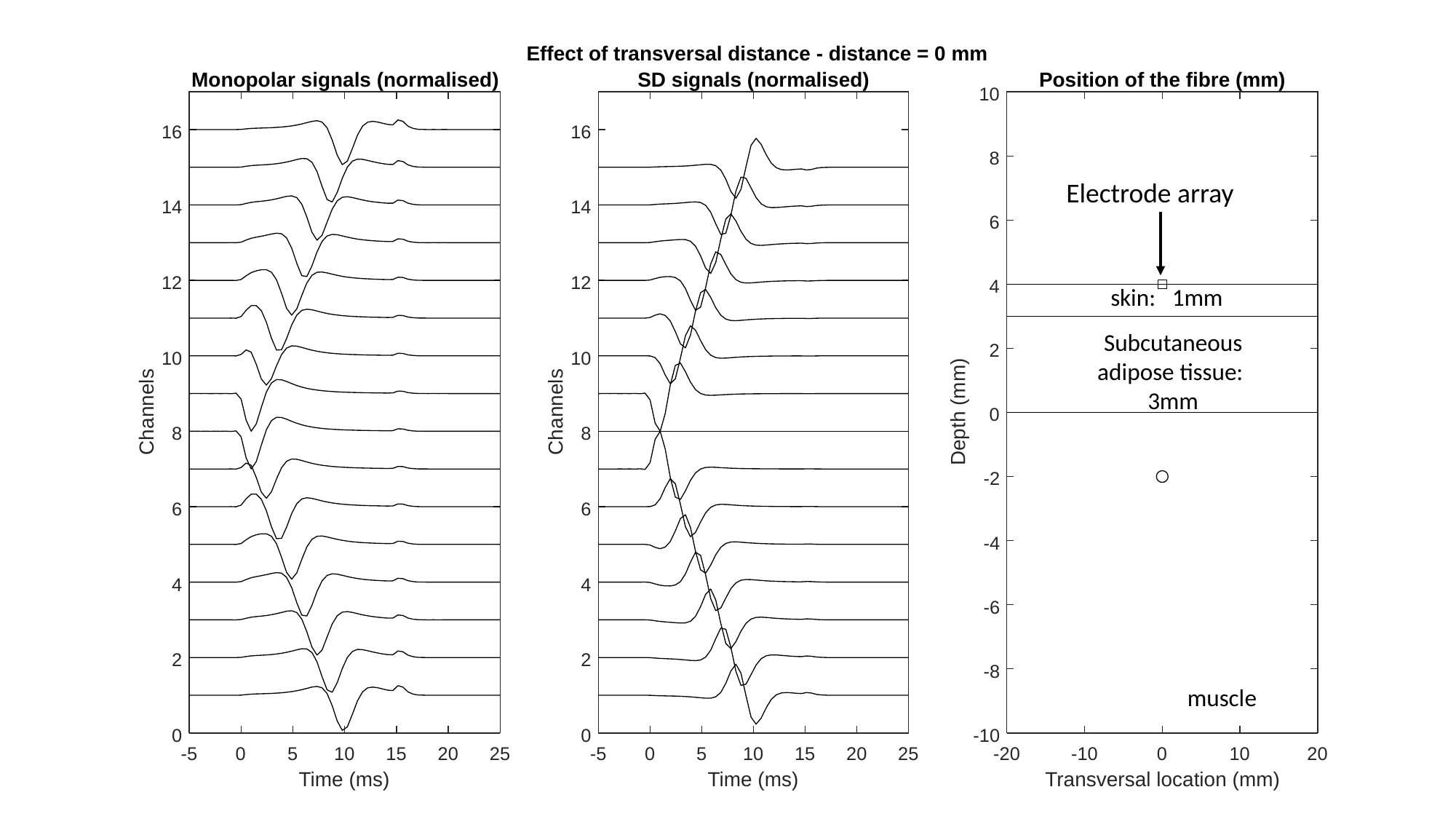

#
Electrode array
skin: 1mm
Subcutaneous adipose tissue: 3mm
muscle

## Slide 3
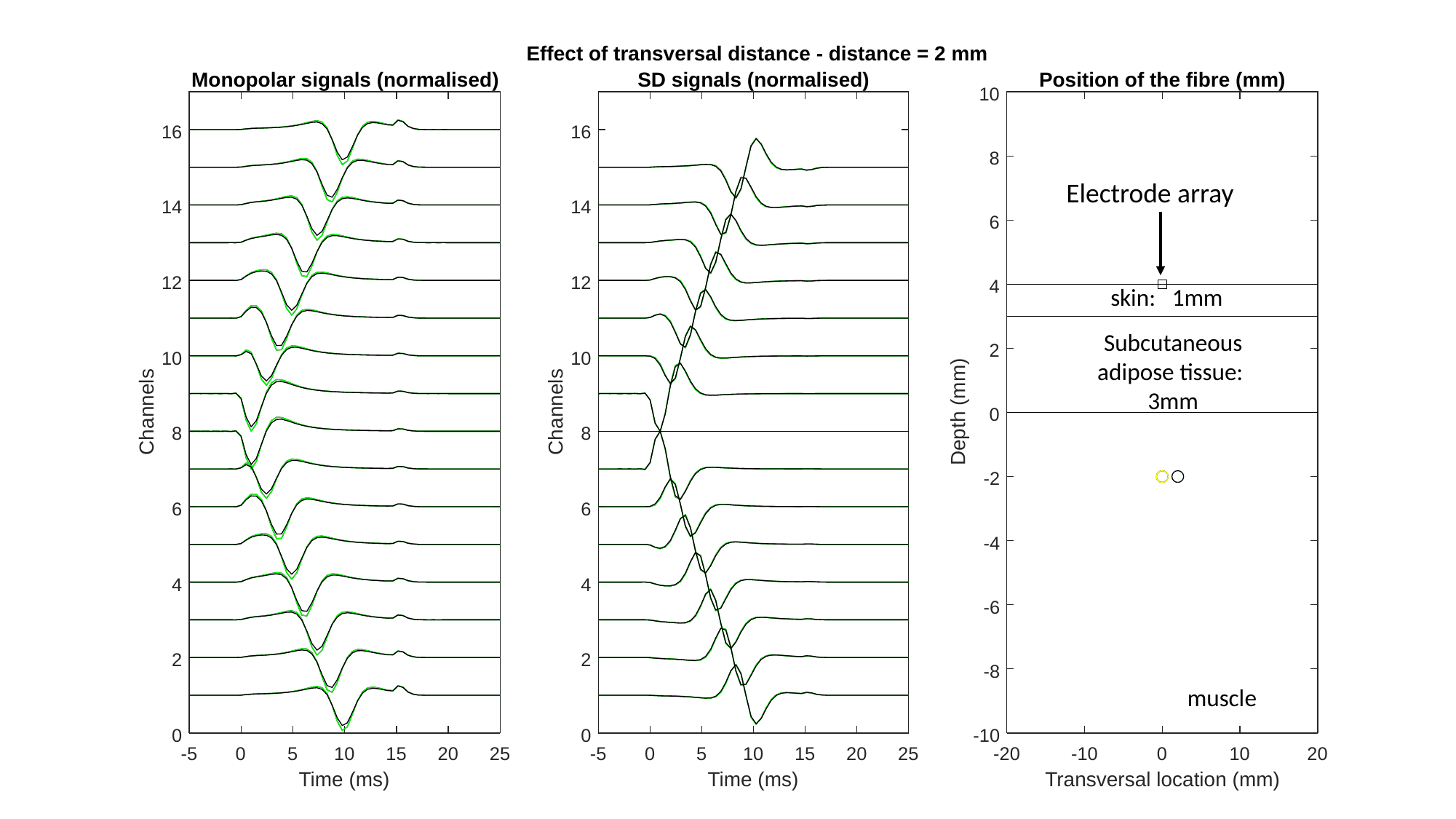

#
Electrode array
skin: 1mm
Subcutaneous adipose tissue: 3mm
muscle

## Slide 4
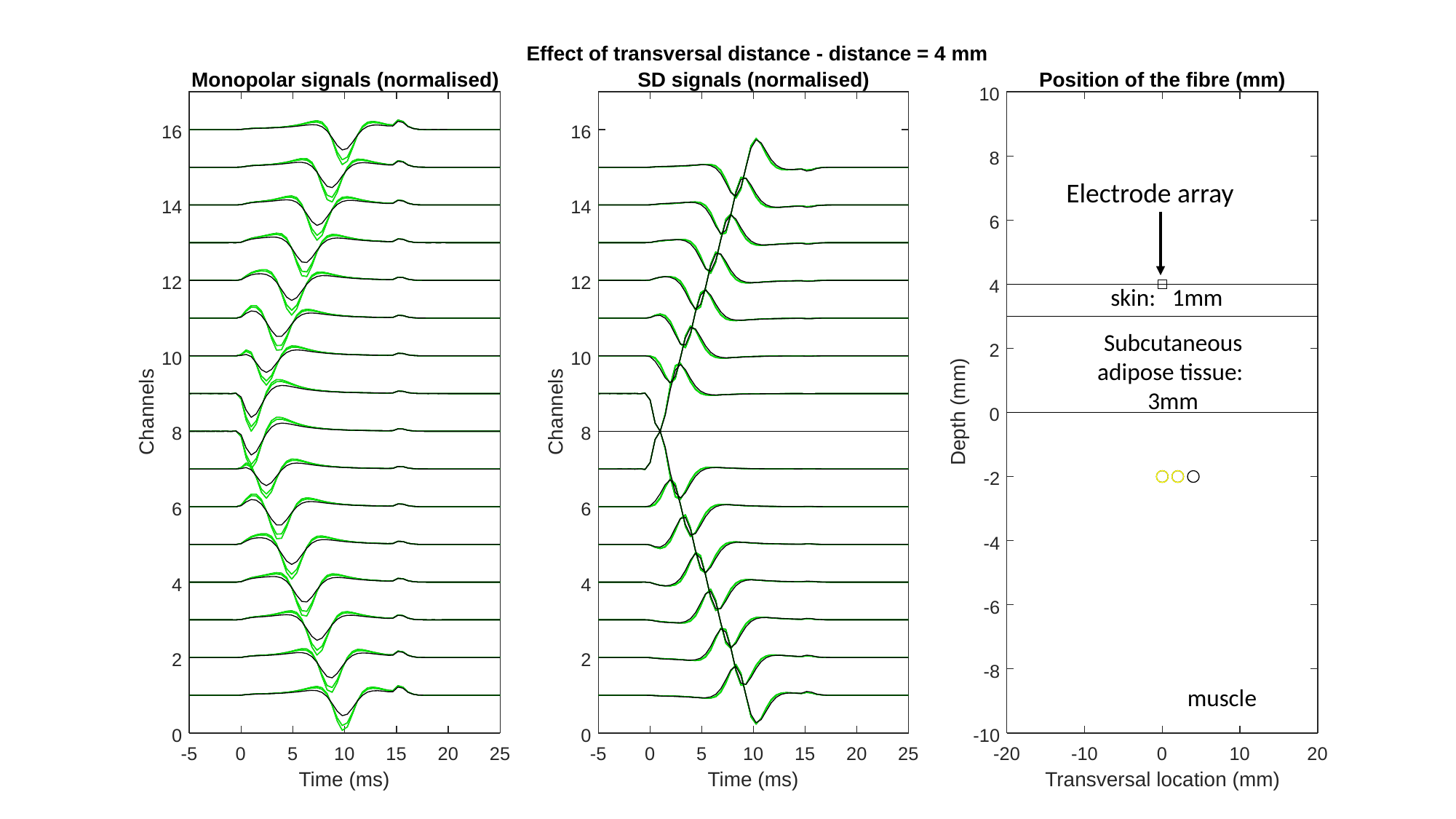

#
Electrode array
skin: 1mm
Subcutaneous adipose tissue: 3mm
muscle

## Slide 5
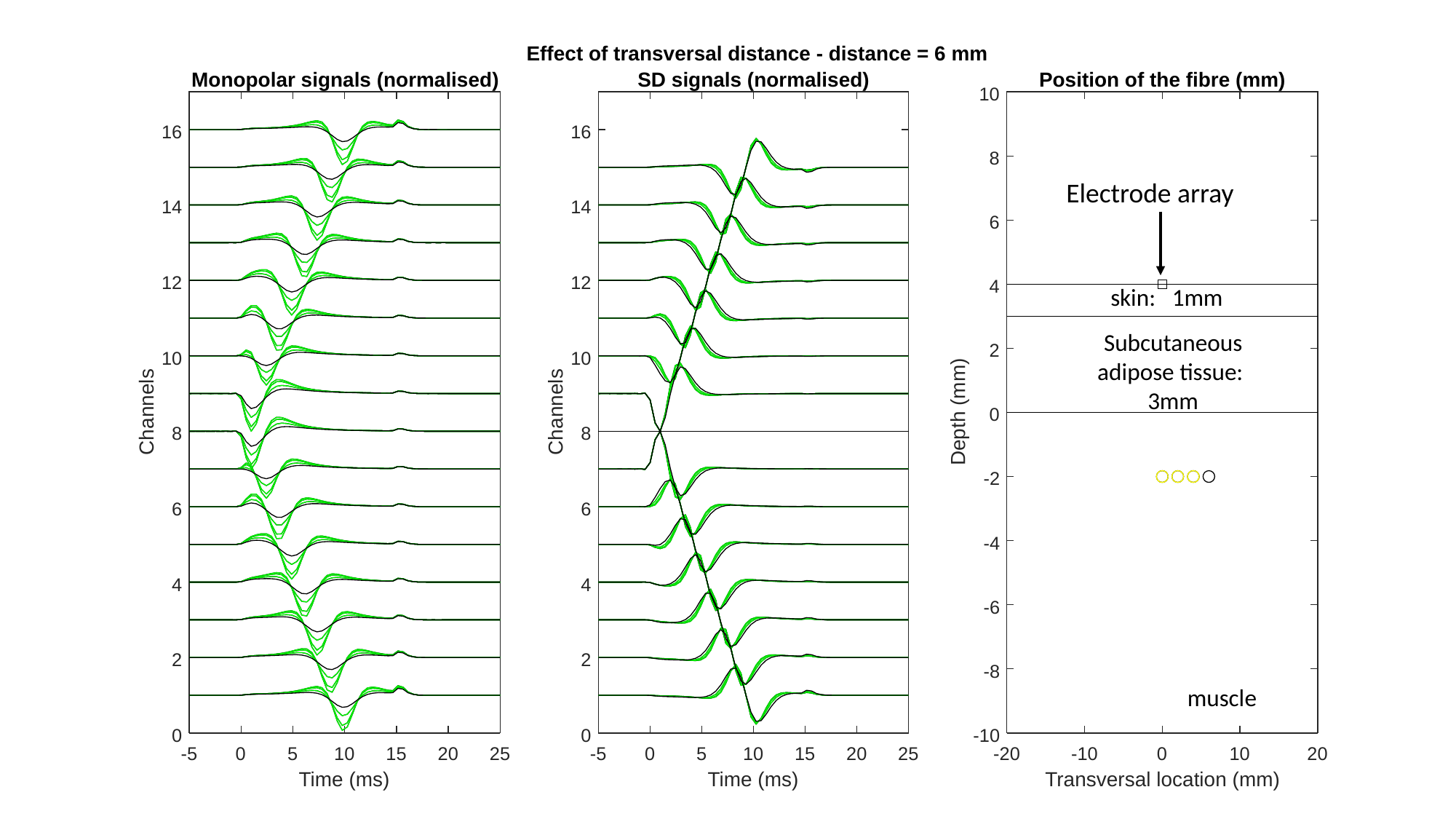

#
Electrode array
skin: 1mm
Subcutaneous adipose tissue: 3mm
muscle

## Slide 6
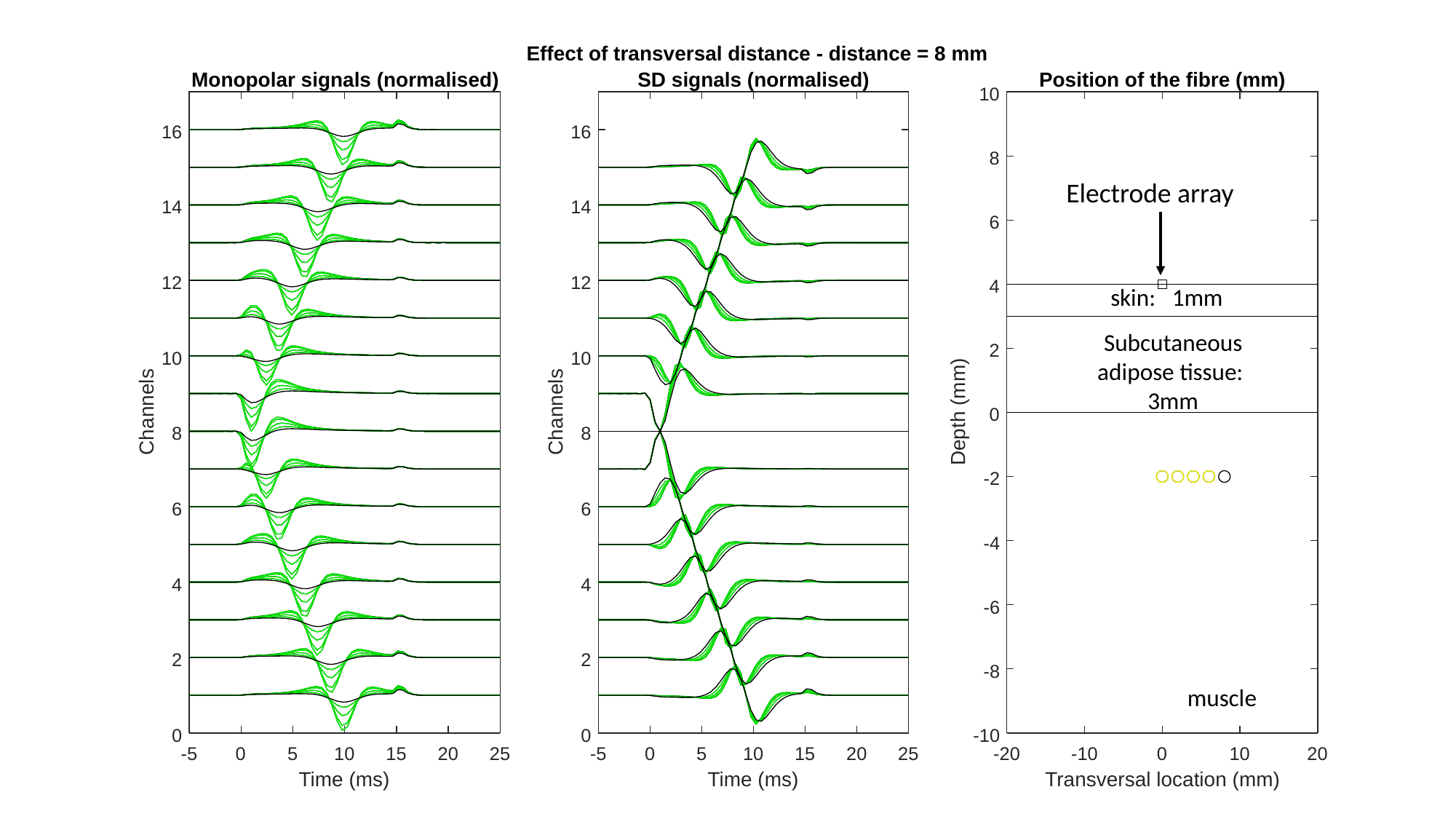

#
Electrode array
skin: 1mm
Subcutaneous adipose tissue: 3mm
muscle

## Slide 7
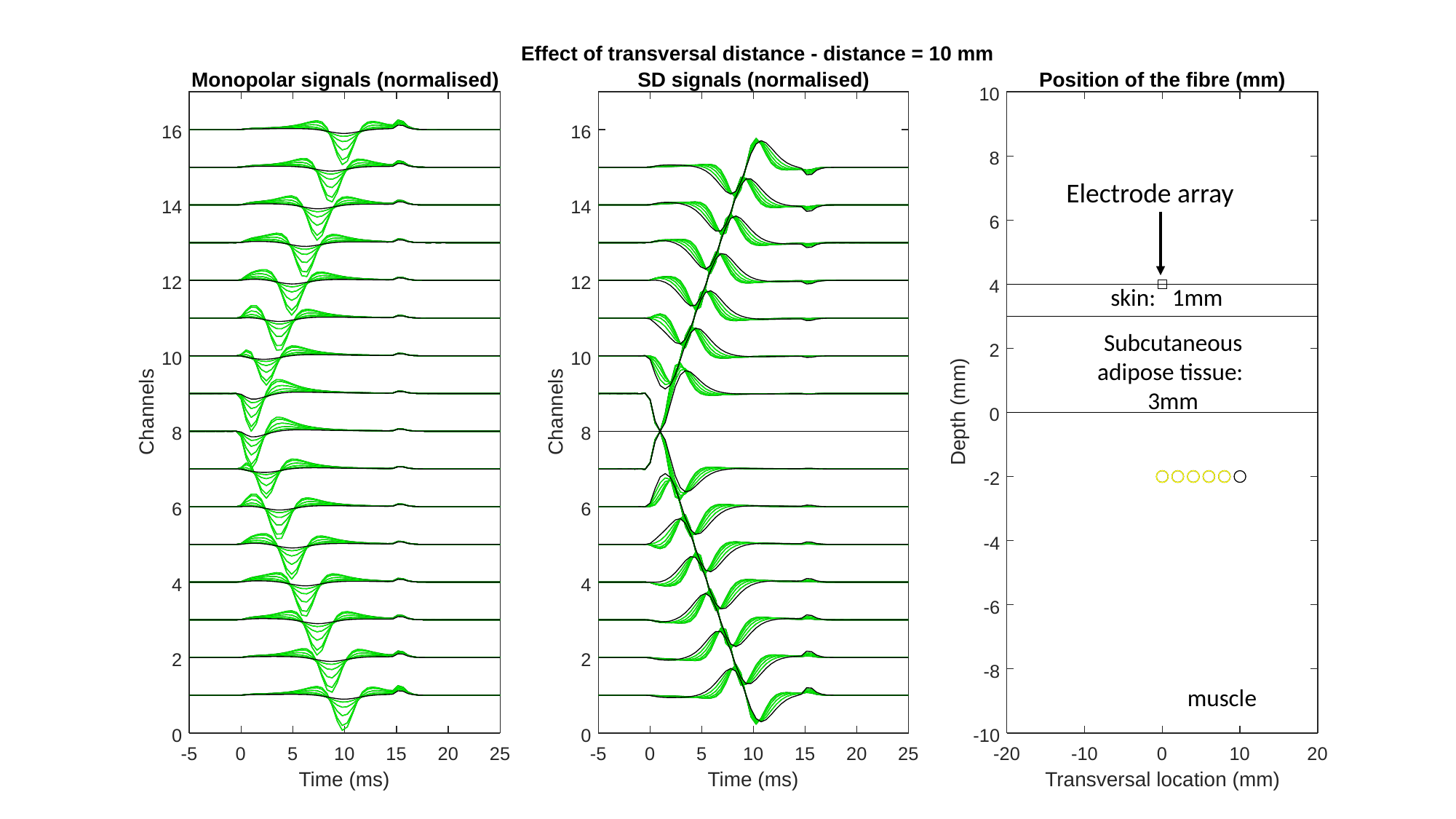

#
Electrode array
skin: 1mm
Subcutaneous adipose tissue: 3mm
muscle

## Slide 8
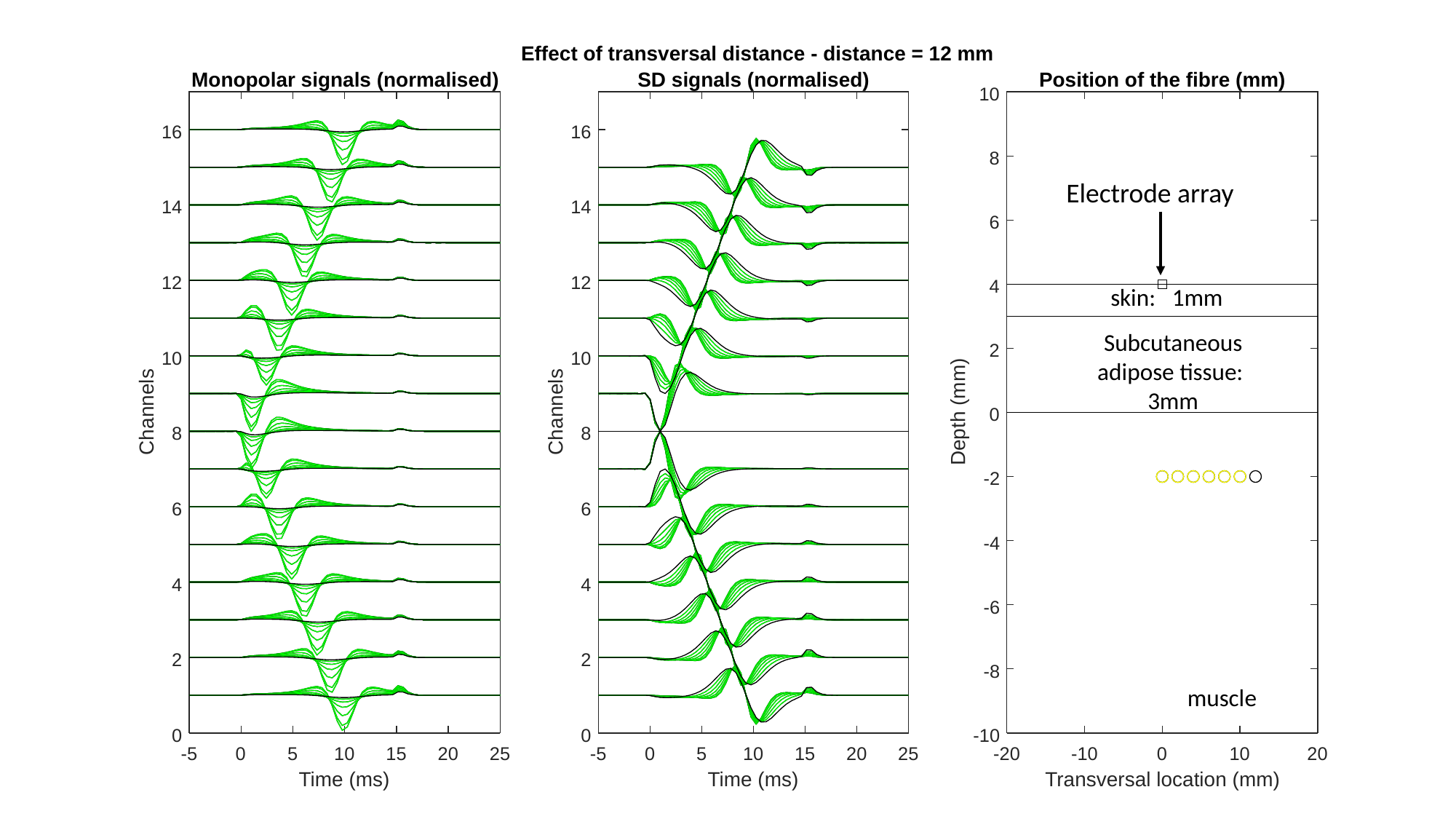

Electrode array
skin: 1mm
Subcutaneous adipose tissue: 3mm
muscle

## Slide 9
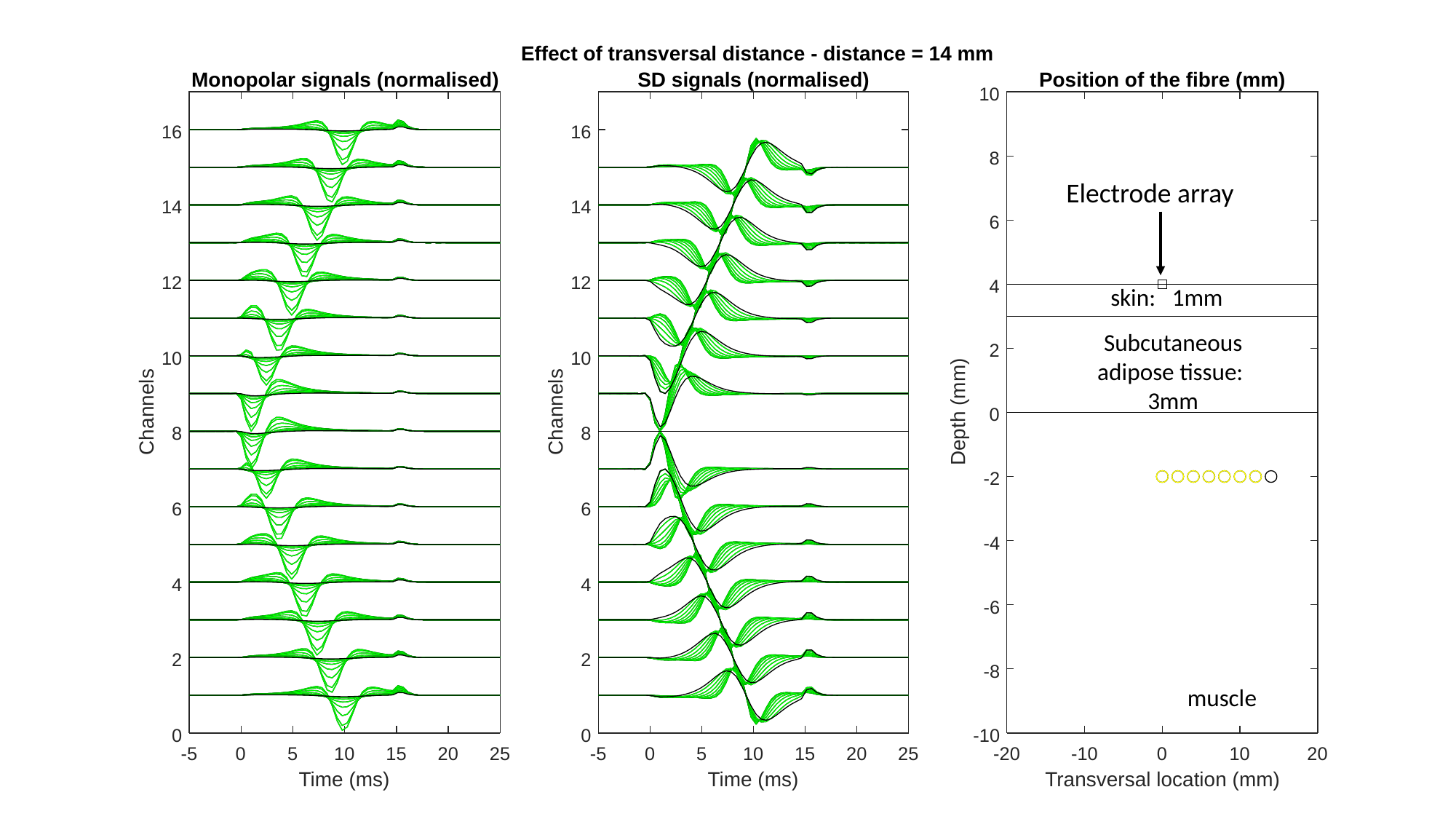

Electrode array
skin: 1mm
Subcutaneous adipose tissue: 3mm
muscle

## Slide 10
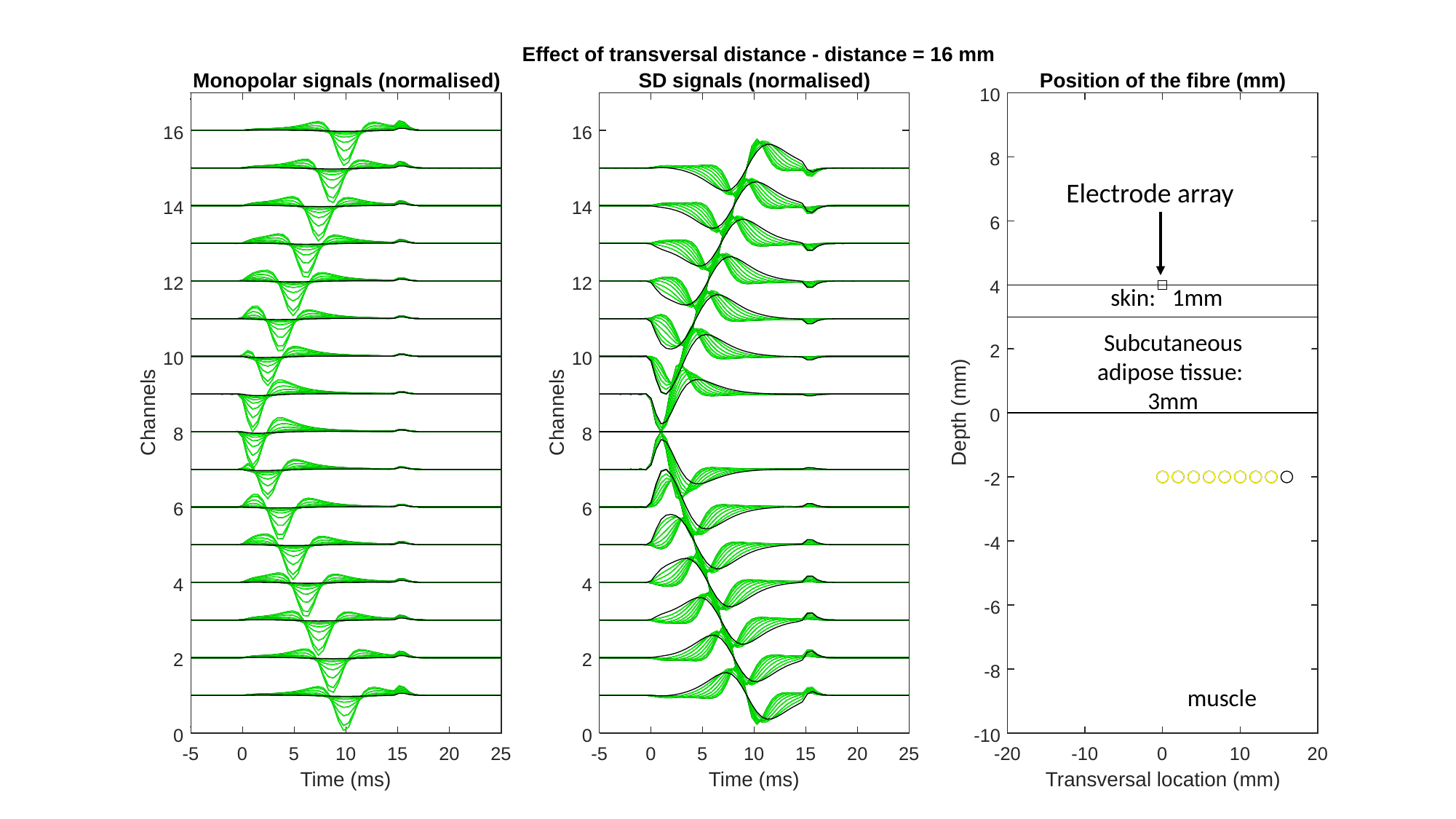

Electrode array
skin: 1mm
Subcutaneous adipose tissue: 3mm
muscle
