# Supplementary figures and images for: Fundamental Concepts of Bipolar and High-Density Surface EMG Understanding and Teaching for Clinical, Occupational, and Sport Applications: Origin, Detection, and Main Errors
Source: Sensors (Basel). 2022 May 30;22(11):4150. doi: 10.3390/s22114150 (PMC9185290; doi:10.3390/s22114150)

## Slide 1
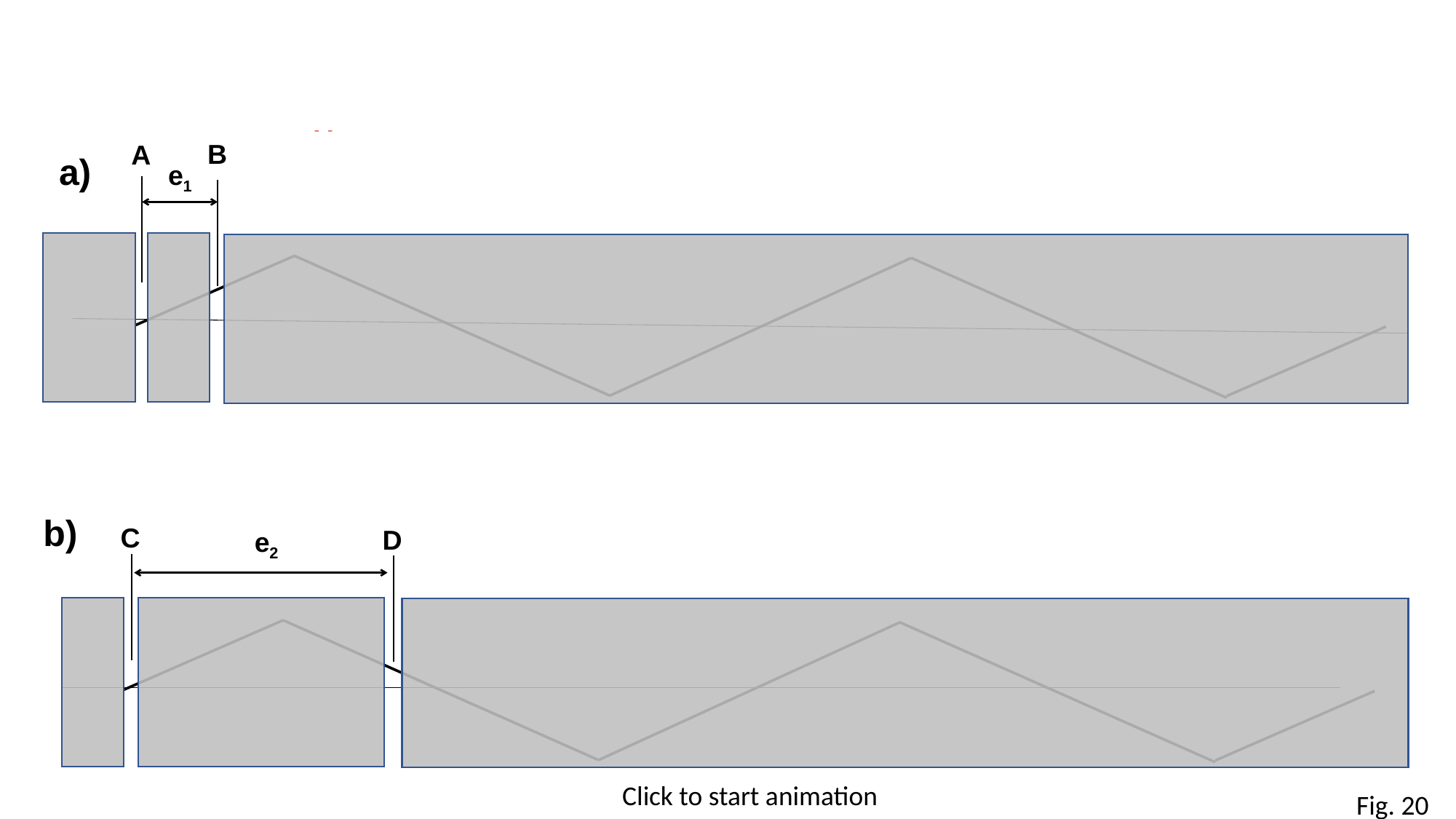

VB - VA
B
A
a)
e1
time
VD - VC
time
b)
C
D
e2
Click to start animation
Fig. 20

Supplement: Supplementary file 1 [file sensors-22-04150-s001.zip › Sup11_Figure_20.pptx]

## Slide 1
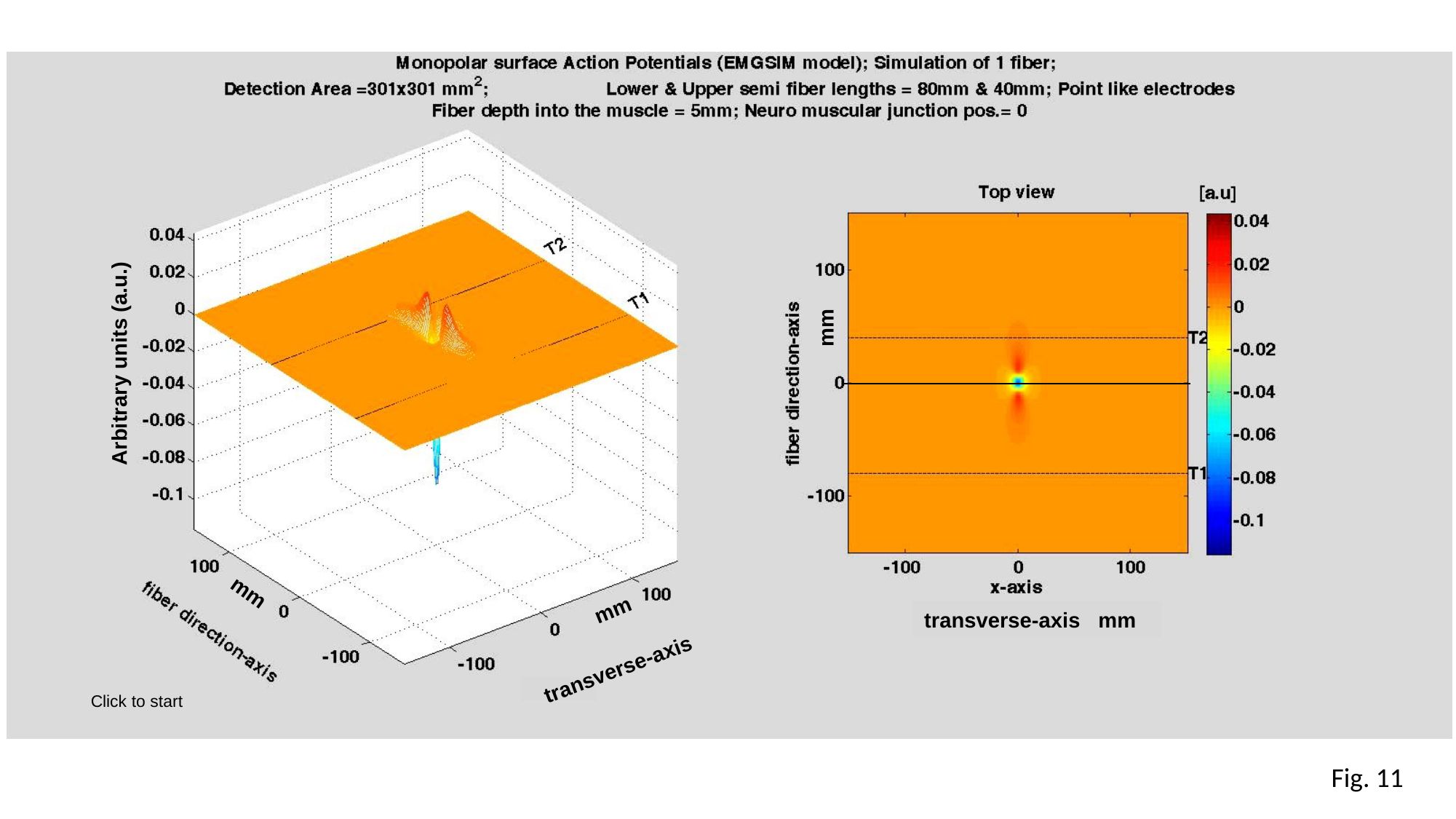

mm
Arbitrary units (a.u.)
mm
mm
transverse-axis mm
transverse-axis
Click to start
Fig. 11

Supplement: Supplementary file 1 [file sensors-22-04150-s001.zip › Sup5_Figure_11.pptx]
